# Thinking about default enrollment lowers vaccination intentions and public support in G7 countries

**DOI:** 10.1093/pnasnexus/pgae093

**Published:** 2024-02-26

**Authors:** Sanchayan Banerjee, Peter John, Brendan Nyhan, Andrew Hunter, Richard Koenig, Blake Lee-Whiting, Peter John Loewen, John McAndrews, Manu M Savani

**Affiliations:** Institute for Environmental Studies, Vrije Universiteit Amsterdam, Amsterdam 1081 HV, The Netherlands; Department of Political Economy, King's College London, London WC2R 2LS, UK; Department of Government, Dartmouth College, Hanover, NH 03755, US; Munk School of Global Affairs & Public Policy, University of Toronto, 315 Bloor Street West (at the Observatory) Toronto, Ontario, M5S 0A7, Canada; Department of Political Economy, King's College London, London WC2R 2LS, UK; Department of Political Economy, King's College London, London WC2R 2LS, UK; Munk School of Global Affairs & Public Policy, University of Toronto, 315 Bloor Street West (at the Observatory) Toronto, Ontario, M5S 0A7, Canada; Department of Political Science, McMaster University, 1280 Main Street West Hamilton, Ontario L8S 4L8, Canada; Munk School of Global Affairs & Public Policy, University of Toronto, 315 Bloor Street West (at the Observatory) Toronto, Ontario, M5S 0A7, Canada; Department of Social and Political Sciences, Brunel University London, Kingston Ln, London, Uxbridge UB8 3PH, UK; Department of Social and Political Sciences, Brunel University London, Kingston Ln, London, Uxbridge UB8 3PH, UK

**Keywords:** nudge, nudge+, reflection, policy effectiveness, policy support

## Abstract

Policymakers often face a conundrum between being transparent about policies and ensuring that those policies are effective. This challenge is particularly relevant for behavioral nudges, which are not usually disclosed. Rather than avoiding transparency, we suggest that policymakers encourage citizens to reflect on nudges to help them understand their own views and align those views with their behaviors. Using data from an online survey experiment with 24,303 respondents in G7 countries, we examine the impact of reflection on a hypothetical default nudge policy for COVID-19 booster appointments. Contrary to expectations, participants say they would be less likely to get the booster when automatically enrolled compared with a control condition. Similarly, encouraging citizens to think about the status quo (baseline) policy also reduces intentions for boosters. These interventions have no effect on approval of the policy. Further, encouraging people to think about automatic enrollment decreases approval of the policy and further decreases their intentions to get vaccinated. These findings suggest that reflection on a nudge can increase backlash from a nudge and also elicit policy disapproval, thereby aligning policy support with behavioral intentions.

**Significance Statement**

Behavioral nudges can effectively encourage citizens to engage in prosocial behavior, but they often operate covertly. To enhance their legitimacy, we propose encouraging the public to reflect on nudges. In a survey experiment conducted among 24,303 participants in G7 countries, we evaluated the effect of reflection in the context of a hypothetical COVID-19 booster appointment default policy. Contrary to expectations, the default reduced vaccination intentions and did not measurably change policy approval. Reflecting on the default exacerbated this negative effect on intentions and also diminished policy support. In this sense, reflection on nudges may help citizens form policy evaluations that align with the behavioral effects of the interventions in question.

## Introduction

Providing explicit explanations and justifications of public policies can sometimes decrease the positive effects of these policies, generating a trade-off between transparency and citizen welfare (1). This dilemma is particularly acute for behavioral policies, like nudges, through which governments attempt to improve the choices made by citizens by altering their “choice architecture” without directly dictating individual actions (2). This style of “libertarian paternalism” (3, 4) has sparked debate about the ethics of policy interventions that shape people's choices without disclosure (5–10). Behavioral nudges are said to differ from traditional “command and control” policies like taxes in their public visibility—often referred to as the publicity principle (11). While most traditional public policies are overt, some nudges, like defaults, seek to alter the choice architecture that individuals face rather than, say, communicating information. The covertness of the nudge facilitates its effectiveness, as exemplified by the phrase that nudges often “work in the dark” (12). Prior research has considered the effects of disclosure either prior to or after a nudge intervention (13–15) but not simultaneously. There is mixed evidence on the impact of disclosure on the effectiveness of the nudge, varying in the type of disclosure used (16, 17).

To address concerns about the potential undue influence of nudges, we evaluate a new type of behavioural public policy intervention called “nudge+” (18), which combines a behavioral nudge with a prompt to think. This intervention seeks to make nudges more legitimate by encouraging people to think about the policy or choice in question and thereby facilitating citizen reflection on nudges. Such an alternate approach can empower citizen autonomy and agency by making individuals watchful of government policies and intentional in their choices and actions (19). Nudge+ builds on prior research suggesting that offering such transparency and reflection may improve the effectiveness of nudges when citizens’ goals are aligned with the nudge (20–22).

Building on studies testing the effects of nudges on vaccination uptake (22–29), in this study, we extended the research on “nudge+” to evaluate its effects on promoting booster vaccine uptake intentions during the COVID-19 pandemic. In an online survey experiment conducted among 24,303 participants in the G7 group of advanced industrialized countries (Canada, France, Germany, Italy, Japan, the United Kingdom, and the United States of America), the participants were randomly assigned to one of four conditions in a 2 × 2 factorial design. Individuals were randomized along two dimensions: default enrollment, in which they would either be automatically enrolled into vaccine booster appointments with local clinics calling to schedule appointments at their convenience or one in which they would make their own appointments; and reflection, in which they were either encouraged to reflect on the government's actions separately or not. This design yielded four conditions: a condition in which participants were presented with a policy in which individuals initiated their own appointments for a booster vaccine (control); a condition in which participants were presented with a policy in which they would be automatically enrolled, by default, to receive a vaccine and the local clinic would contact them to schedule appointments at their convenience (nudge); a condition in which participants were presented with the control condition and then asked to reflect on it (think); and a condition in which participants were presented with the nudge condition and then asked to reflect on it (nudge+). We considered the effects of these interventions on two outcomes: vaccination intention for the booster and approval of the government's actions.

Contrary to prior research (23, 24, 30, 31), we found that a hypothetical policy of default enrollment into scheduled vaccine appointments produced a backlash, reducing people's behavioral intentions to get the vaccine for themselves. However, approval of this policy did not measurably differ from the status quo in the control condition. Further, when participants assigned to automatic enrollment were prompted to think about the policy, they were even less likely to say they would get a vaccine and their approval of the policy correspondingly decreased. Based on these findings, we concluded that a hypothetical default opt-out nudge did not increase the reported willingness to get a COVID-19 vaccine booster. Our findings contradict common assumptions about the power of a (hypothetical) default nudge to increase vaccinations (32) and add to a growing (mixed) evidence base for using defaults to influence vaccination outcomes (33).

We make two important contributions to the growing literature in behavioral science and public policy. First, our experiment evaluates the effects of a range of interventions considered in behavioral public policy, such as nudges, thinks, and nudge+, in the context of a timely public policy issue. Second, our findings suggest that nudge+ can reconcile the trade-off between effectiveness and support—encouraging citizens to reflect on a default enrollment policy diminishes public support for what turns out to be an ineffective nudge, suggesting that reflection may help people better align policy approval of nudges with their behavioral consequences and thereby provide a valuable signal to policymakers.

## Experimental design

### Survey design

We administered a preregistered online survey experiment to 24,303 respondents in Canada, France, Germany, Italy, Japan, the United Kingdom, and the United States of America. The sample size was selected based on the power analysis reported in the Appendix. The survey was administered on Qualtrics to national samples that were representative by age, gender, education, and subnational region (for summary statistics, see Tables S2–S11) by *Dynata* from January 27 to February 26 in 2022. Table S12 provides country-specific date ranges for the periods in which surveys were fielded within this interval. Respondents were paid at standard rates recommended by Dynata. The original survey was written in English and localized into French, German, Italian, and Japanese languages by translators at Dynata, which were then cross-validated by first-language speakers. The study preregistration is available online through Open Science Framework (OSF). The English (United Kingdom) version of the survey is provided in the Appendix, and all versions of the surveys are also available online.

### Experimental vignettes

We used a between-subjects experimental design with four different treatment conditions, including the control. In each condition, respondents were presented with a hypothetical scenario taking place in October 2022 in which “COVID-19 cases are rising in your area” and “[t]he government is making another vaccine booster shot freely available to you as winter is approaching.”

Following this information, respondents were randomized into four different experimental vignettes that are described in Table 1. The experimental conditions can be expressed as a 2 × 2 factorial design in which individuals are either automatically enrolled into receiving a booster vaccine, with local clinics calling them to schedule appointments at their convenience, or make their own appointments (default enrollment) and are either encouraged to reflect on the government's actions or not (reflection). Table 2 shows this 2 × 2 factorial design. The treatment conditions can be expressed as combinations of these dimensions: control (default enrollment = no, reflection = no), nudge (default enrollment = yes, reflection = no), think (default enrollment = no, reflection = yes), and nudge+ (default enrollment = yes, reflection = yes). For example, respondents in the control condition were told that people who wanted a booster would have to schedule an appointment. Respondents in the nudge condition were told that they would be automatically enrolled, by default, to receive a vaccine and the local clinic would contact them to schedule appointments at their convenience. We designed this nudge to be as flexible as possible to minimize opt-outs of the default enrollment due to scheduling conflicts. Respondents in the think condition were provided with an open-text question asking them to reflect on whether the government's policy was appropriate and would work for them. These questions were chosen to first de-bias the participants of any undue influence of the vaccine policy—debiasing individuals, in this way, has been shown to help citizens in articulating their true preferences (see, e.g. Fischoff (34), and Milkman et al. (35))—and then build their agency by empowering them to evaluate the goals of the nudge. Respondents who wrote fewer than 75 English characters (or equivalent in other languages as preregistered; see Table S20 for details) in the think condition were asked to write more. Finally, those in the nudge+ condition were told of the default enrollment policy and then asked to reflect about it in an identical manner to those in the think condition. Since the interpretation of the term “enrollment” can vary in different contexts such as the different G7 countries, we provide country-level versions of the results in Tables S17 and S18.

**Table 1.** Text of experimental vignettes.

Treatment	Vignette description
Control	In this scenario, the government leaves it to every adult living in your country to choose whether they should get this vaccine booster shot or not. If you want a booster, you will have to call your local clinic to schedule a booster appointment.
Nudge *Default enrollment*	In this scenario, the government announces that every adult living in your country will be automatically enrolled to receive this vaccine booster shot at a local clinic. Your local clinic will call you to schedule a booster appointment at a convenient date and time. You can opt out of this automatic enrollment if you wish.
Think *Reflection*	In this scenario, the government leaves it to every adult living in your country to choose whether they should get this vaccine booster shot or not. If you want a booster, you will have to call your local clinic to schedule a booster appointment. Please think about the government's actions in this scenario. Do you think this approach is appropriate? Do you think this approach will work for you? In at least one or two sentences, please write down your thoughts. [text box]
Nudge+ *Default enrollment and reflection*	In this scenario, the government announces that every adult living in your country will be automatically enrolled to receive this vaccine booster shot at a local clinic. Your local clinic will call you to schedule a booster appointment at a convenient date and time. You can opt out of this automatic enrollment if you wish. Please think about the government's actions in this scenario. Do you think this approach is appropriate? Do you think this approach will work for you? In at least one or two sentences, please write down your thoughts. [text box]

**Table 2.** Experimental design.

		Default enrollment No Yes
Reflection	No Yes	Control Nudge Think Nudge+

Respondents were then asked the following outcome measures:

- Intentions to get the booster dose on a 6-point scale from very unlikely (1) to very likely (6).
- Approval of the actions of the government on an 11-point scale from “I disapprove of the government's action” (0) to “I approve of the government's action” (10).

The first outcome measures participants’ stated intention to accept the booster vaccine and the second outcome measures support for the policy. Due to the self-reported nature of our survey experiment, we are unable to measure real vaccination behaviors. To measure respondents’ compliance with the experimental vignettes, we used a preregistered manipulation check to assess their recall of the vaccine policy shown in their condition. Respondents were asked “what did the government do to manage rising COVID-19 cases in your area?” in the scenario and asked to choose among four choices, including default appointment scheduling and self-directed scheduling (for exact wording, see the questionnaire provided in the Appendix). The third option in our manipulation check question had a typo. It stated that “The government announces that every *living adult* in your country…” instead of “The government announces that every *adult living* in your country…” This error was consistent across all treatment conditions.

We preregistered the hypotheses that being assigned to the nudge or nudge+ conditions would improve people's intentions to get vaccinated (H1a and H2a, respectively) and approval of the government's policies (H1b and H2b, respectively) vs. the control condition. Further, we also preregistered that the nudge+ intervention would increase the effects of the nudge (H3a) and public approval of the policy (H3b) vs. the nudge condition. These hypotheses follow Banerjee and John (18), who theorize that spurring people to think about a nudge enables them to assess its merits and evaluate it with respect to their own goals. If those goals are aligned with the nudge (on average), then uptake of the nudge should increase. Support for the policy may also increase as well due to the transparency of this approach. For further details on our theoretical reasoning, see the Appendix.

## Methods

Our experimental design, protocol, and methods were approved by the research ethics board of King's College London and the London School of Economics and Political Science. Informed consent was obtained from all participants prior to their participation. All methods were performed in accordance with the relevant guidelines and regulations.

We test our preregistered hypotheses using Ordinary Least Square (OLS) models with robust SE in which we regress behavioral intentions to get the booster vaccine dose and approval of the government's actions on indicators for the experimental treatments (nudge, think, and nudge+). As preregistered, each model includes country-fixed effects as well as covariates selected using the lasso to increase the precision of our treatment effect estimates (36). These models estimate intent-to-treat (ITT) effects. All results are unweighted. An exploratory analysis of the ITT effects of the experimental conditions, expressed as a 2 × 2 factorial design, in which we measure the effect of interaction between default enrollment and reflection, is provided in Table S19.

However, we find that the receipt of treatment is often low, as described below. We, therefore, follow our preregistration in also estimating complier average causal effects (CACEs) using two-stage least-squares models in which we use random assignment as an instrument for the following measures of treatment compliance:

- Nudge condition compliance: 1 if respondent assigned to the nudge condition and answers the manipulation check question about it correctly, 0 otherwise.
- Think and nudge+ compliance: number of sentences written if assigned to the condition in question and answers the manipulation check question about it correctly, 0 otherwise. For think and nudge+ compliance, we also use two alternative compliance specifications: we standardize the number of sentences by country (exploratory) or take the square root of the number of characters written by respondents (preregistered). The results of these alternative specifications are available in Table S16.

We also control for selected lasso covariates and country-fixed effects and use robust SEs as in the models described above. For all models estimated below, our inference is based on randomization-*t p*-values (37). We did not preregister any ex-post multiple hypotheses correction method. Instead, we incorporated a conservative Bonferroni correction into the power calculation used to select our sample size (see Appendix for details). We use Stata 17.1 to conduct statistical analyses and the Quanteda package in R for text analysis.

## Results

**Fig. 1.**
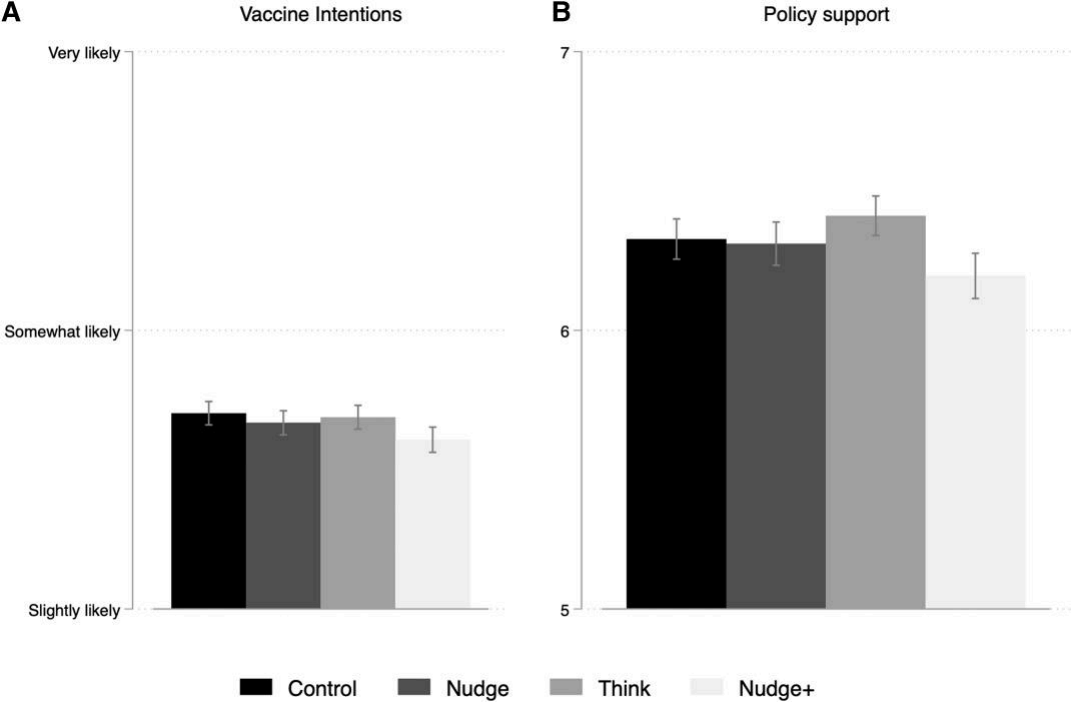
The CI bar plots of A) intentions to get the booster dose and B) approval of actions of the government.

The resulting experimental data satisfies our preregistered balance tests and shows expected levels of demographic diversity (see Appendix for details and summary statistics). Overall, respondents’ intentions to get the booster dose for themselves are generally high across all conditions (mean of 4.7 on a 6-point Likert scale). Respondents’ policy approval is centered around the midpoint of the scale (a mean of 6.3 on an 11-point scale). We begin our analysis with Fig. 1 which shows the mean values and 95% CIs for the two outcome measures across the four different experimental conditions.

Next, Table 3 presents ITT effects on vaccination intentions and approval of policy. Contrary to our expectations, the nudge intervention of default vaccination enrollment reduced respondents’ intention to get a booster dose by 0.065 units on a 6-point scale (*P <* 0.005)—in other words, respondents who were nudged into a default enrollment were 0.016 SDs less likely to accept the vaccine compared with those who were left to schedule their own booster vaccination. The think condition also produces a negative effect on behavioral intentions to get a booster (−0.058 or −0.014 SD, *P <* 0.005).^a^ In contrast, defaulting people into vaccine enrollments or encouraging them to reflect on the vaccine policy produces no measurable effect on approval vs. self-scheduling in the control condition (nudge = 0.037, n.s.; think = 0.035, n.s.). Most importantly, encouraging respondents to reflect on the default appointment policy in the nudge+ condition further decreased intentions to vaccinate relative to the negative effect observed in the nudge condition (−0.125 or −0.031 SD, *P <* 0.005 vs. controls; −0.059, *P <* 0.005 vs. nudge; −0.066, *P <* 0.005). Due to a coding error, respondents were required to answer the approval question in the nudge+ condition but not in other conditions. However, missingness was <1% in the control (48 responses), nudge (44 responses), and think (3 responses) conditions, and our ITT results are robust to randomly dropping 1–5% of the nudge+ observations in percentage-point intervals (see Tables S14 and S15). Nonetheless, the nudge+ intervention also reduced policy approval (−0.150 or −0.021 SD, *P <* 0.005 vs. controls; −0.112, *P <* 0.05 vs. nudge; −0.184, *P <* 0.005). Findings from the exploratory analysis, in which we respecify the model as an interaction between default enrollment and reflection, are reported in Table S19 and are equivalent to those reported in Table 3.

**Table 3.** ITT effects on vaccination intentions and policy approval.

	Intentions	Approval
Nudge	−0.065^a^	−0.037
	(0.020)	(0.046)
Think	−0.058^a^	0.035
	(0.020)	(0.049)
Nudge+	−0.125^a^	−0.150^a^
	(0.021)	(0.048)
Controls	✓	✓
Country FE	✓	✓
*N*	24,164	24,115

OLS estimates with robust SEs in parentheses. ^a^*P*  *<* 0.005 (Young 51 randomization-*t P*-values). Controls selected by lasso linear regression specification. Column 1 includes controls for age, gender, parental status, town/city type, religious beliefs, prior COVID-19 infection status (self), vaccination status, booster status, and trust in vaccines (binary). Column 1 retains all nudge+ observations. Column 2 includes controls for age, gender, parental status, religious beliefs, prior COVID-19 infection status (self), booster status, and trust in vaccines (binary). FE (fixed effects).

We examine compliance rates to see whether respondents received the treatment as intended. We find that manipulation check passage rates by condition vary between 45% (nudge+) and 69% (nudge), indicating that many respondents were unable to comprehend fully the government policy in question.

Noncompliance was statistically uncorrelated with respondent inattention in a preregistered attention check (*F* = 0.40, *P* = 0.75; see Appendix for question wording). An exploratory analysis of compliers following Marbach and Hangartner (38) shows that compliance with the nudge or think is not significantly associated with respondent gender, parenthood, city/town type, religious beliefs, prior COVID-19 incidence, trust in vaccines, or prior vaccine and booster uptake. However, we find that adults without children and those who live in smaller towns/cities are more likely to successfully receive the nudge+ treatment (see Fig. S4). We, therefore, follow our preregistered approach to estimate CACEs. These models use indicators for random assignment as instruments for endogenous measures of treatment receipt. For nudge, the endogenous measure of treatment receipt is answering the manipulation check question correctly. For think and nudge+, we use the number of sentences written in the open-text prompt (either as an integer or an exploratory measure standardized by country) and the square root of the total number of characters written standardized by country. A more detailed analysis of the textual responses is provided in the Appendix (see the Text analysis section).

**Fig. 2.**
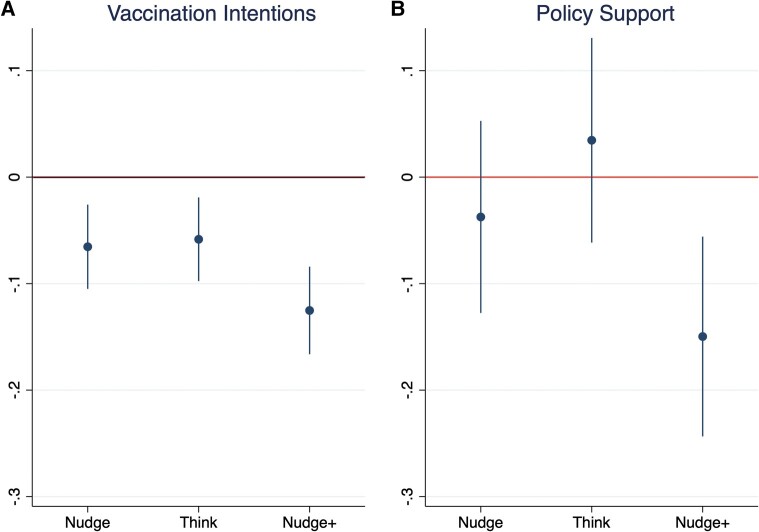
Coefficient plot of ITT effects for A) intentions to get the booster dose and B) approval of actions of the government.

The main effects of treatments among compliers, which are reported in Table S16, are consistent with the ITT estimates in Table 3 across instrumental variable specifications. The nudge, think, and nudge+ treatments all reduce booster vaccination intentions relative to the control condition. As in the ITT analysis, the effects on approval are null for the nudge and the think, and negative for the nudge+ vs. the control condition. The nudge+ consistently lowers vaccination intentions and policy approval vs. the nudge and the think conditions. These ITT effects (controlling for variables selected by the Least Absolute Shrinkage and Selection Operator (LASSO), and country fixed effects) are shown in Fig. 2.

Finally, as preregistered, we conduct exploratory checks for the robustness of these treatment effects across each country, which are reported in Tables S17 and S18. The negative effects on booster intentions in the pooled sample are statistically detectable in the United States and Germany for nudge; the United Kingdom and Japan for think; and all countries but France and Italy for nudge+. The nudge+ condition does not significantly increase the backlash effect of the nudge in any country. Further, we find the nudge measurably increases policy approval vs. the control condition in the United Kingdom and Japan, while decreasing it in the United States. The think increases policy approval vs. the control in the United States and France, while it decreases it in the United Kingdom and Italy. The nudge+ decreases approval vs. the control condition in every country, except Italy, where we find a null, and the United Kingdom and Japan, where we see a positive effect. There is no statistical evidence to suggest that the nudge, think, or nudge+ conditions increase vaccination intentions in any G7 country vs. the status quo in the control condition. All within country effects on vaccination intentions are broadly consistent with our pooled findings in Table 3. We also report exploratory heterogeneity in estimated treatment effects in the Appendix (see Table S21 and Fig. S5).

## Discussion and conclusion

We present experimental evidence on the role of reflective transparency in behavioral public policy (18). Contrary to expectations, we find that a hypothetical default opt-out nudge does not increase survey respondents’ willingness to get a COVID-19 vaccine booster, and support is even lower when they are actively asked to reflect on the policy. In other words, reflecting on the nudge diminishes approval, which better aligns policy approval with intended behavior under the influence of the nudge. These findings suggest that reflective transparency may help citizens think through government actions and generate more informative signals about policy efficacy and likely compliance. For instance, future research should test whether reflecting on a nudge that successfully changes intended behavior (unlike what we find here) generates increased approval for the policy.

Our findings generate insights on how to most effectively use experimentation with citizen feedback in developing behavioral public policy. For example, we contribute to conversations around open, democratic governments as “laboratories for policy experimentation” (39, 40) that search for better policies (41). Our research suggests that citizen reflection might inform “test-learn-adapt” approaches to behavioral policy development (42, 43), while avoiding public reactance (44). Specifically, policymakers can use nudges that encourage citizen reflection to avoid false signals of public support for policies that are likely to be rejected by the public.

In the context of vaccines, default appointments represent an interesting case as they are not fully coercive yet seek to shape people's behavior. Reflection on such a policy can therefore lead to different policy outcomes, as we show. A nudge+ enables policymakers to ascertain underlying preferences when there is an opt-out that might otherwise be disguised. Without this moment of active reflection, policymakers might be puzzled at citizen reactions to opt-out approaches. However, we strongly caution that we are unable to test whether the effects we observe on behavioral intentions would translate into real-world behaviors, which should be validated in future studies if such an approach were undertaken (to date, democratic governments have not sought to automatically enroll people in COVID-19 vaccines).

Our findings on the negative effects of a hypothetical default nudge also contribute to a wider debate on the extent to which nudge effects are sensitive to context. Serra-Garcia and Szech (23) and Tentori et al. (24) find, for instance, that defaults can increase vaccination intentions and behaviors in samples from the United States and Italy, respectively. Consistent with prior evidence suggesting that defaults can fail (44), we observe negative effects of automatic scheduling of hypothetical booster appointments on vaccination intentions. In some cases, nudges like these might be seen as intervening too aggressively; related work finds backlash effects of nudges with organ donation, for instance (45). Practitioners should be more attentive to how to use nudges given the context of the social problem and seek to use reflection as a tool to generate policy signals from citizens.

Further research should assess the external validity of these findings in other times and contexts. It is possible that our findings were influenced by the specific design of the nudge (which could not create a true default in the same manner as a real-world nudge) and the nature of the reflection task. Prior research suggests that treatment effects may vary by the types of disclosures (16) and frames of evaluation (46) used. Further, nudge+ interventions may also differ in the type of reflection embedded in the nudge (47). Heterogeneity in the uptake of these interventions by different target populations should also be studied. For example, our exploratory analysis suggests that male participants, people without a booster, and those who are less trusting in institutions and more right-leaning are less likely to have a positive reaction to nudge+, for both vaccination intentions and policy approval.

Several limitations of our study must be noted. First, we note that the magnitude of our estimated effect sizes is small (0.02–0.03 SDs). Second, we cannot measure the effects of actual nudge policies on vaccination behavior; future studies should extend this research to test the effects of nudge+ interventions in real-world settings before scaling up nudge policies, which can have negative effects. Third, our exploratory findings showing heterogeneity across countries should be investigated further. The effects of reflection can also vary with other nudges. The deployment of our proposed nudge+ policy can be logistically and financially challenging. Further research is required on how to most cost-effectively encourage reflection in the public effectively (see Keppeler et al. (22) who recently deployed a nudge+like mechanism in Germany to improve vaccination behaviors). Fourth, our study took place after the peak pandemic but during a period in which the public was still worried about COVID after the Omicron variant. Further research should assess the external validity of these findings in other times and contexts. Finally, our study is based on cross-sectional data; future research should consider how these vaccination behaviors and policy effects change over time. Despite these limitations, we believe that our findings are novel and informative for future tests of nudge+ interventions.

## Supplementary Material

pgae093_Supplementary_Data

## Data Availability

All data used for this analysis are included in the manuscript and Supplementary material.

## Note

a Note that this effect is significant in the UK and Japan but not in the US, Canada, France, Germany, or Italy.

## Preprints

A previous version of this manuscript was posted as a preprint at https://doi.org/10.21203/rs.3.rs-1964391/v1.
