# Supplementary material for: Thinking about default enrollment lowers vaccination intentions and public support in G7 countries
Source: PNAS Nexus. 2024 Feb 26;3(4):pgae093. doi: 10.1093/pnasnexus/pgae093 (PMC10997051; doi:10.1093/pnasnexus/pgae093)
Supplement: pgae093_Supplementary_Data [file pgae093_supplementary_data.pdf]

Thinking about default enrollment lowers vaccination intentions and public support in G7 countries

Sanchayan Banerjee[*†], Peter John[‡], Brendan Nyhan[§], Andrew Hunter[‡], Richard Koenig[‡], Blake Lee-Whiting,[¶] Peter John Loewen,[¶] John McAndrews[‖], and Manu Savani[**]

[*]Corresponding author: S.Banerjee@vu.nl. Vrije Universiteit Amsterdam, Netherlands. Contact: +31(0)647778058

[†]Vrije Universiteit Amsterdam, Netherlands

[‡]King's College London, United Kingdom

[§]Dartmouth College, United States

[¶]University of Toronto, Canada

[‖]McMaster University, Canada

[**]Brunel University London, United Kingdom

Online Appendix

Research design

Our survey was divided into different parts as described in Figure S1. At the beginning, respondents were informed about the study objectives and then asked for their explicit consent. Consenting respondents were screened out, without affecting our sampling quotas, if they failed an attention check, were under age 18, or were not a resident of the country in question. Respondents then self-reported their vaccine preferences, prior COVID-19 medical history and vaccination status, and social and demographic measures such as political preferences, risk aversion, and trust in different economic agents. Respondents were randomized into a separate experiment that will be reported in a separate paper (see questionnaire below) before being independently randomized into the current experimental design. All randomizations were conducted in Qualtrics. In the current experiment, respondents were randomized into one of four different experimental vignettes. Each vignette was designed to represent a behavioural public policy intended to promote the uptake of a COVID-19 vaccine booster dose. For example, in the *control* condition, the government left it to the public to make appointments to get vaccinated, which is intended to mirror the status quo. In the *nudge* condition, respondents were defaulted into receiving the booster dose through automatic appointment scheduling, a standard nudge intervention that seeks to change behavior by altering defaults. The *think* condition was similar to the *control* except that respondents were asked to reflect on whether the government's approach was appropriate and would work for them, which we anticipated would encourage them to think carefully and make their own decision. Finally, in the *nudge+* condition, the default in the nudge condition was combined with the think prompt encouraging people to reflect on the government's actions, a less paternalistic approach than a nudge intervention alone. These experimental vignettes are detailed in Table 1 in the main text.

Hypotheses

We preregistered the following hypotheses. First, based on encouraging prior research on nudges [42, 33, 45, 46], we hypothesised the following positive effects of the nudge and the nudge+ on vaccination intentions and policy approval:

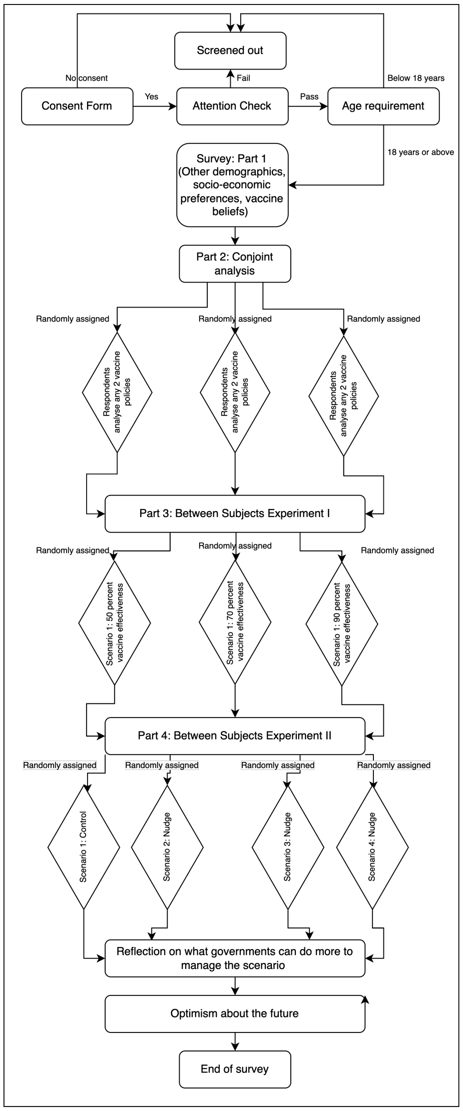

Figure S1: Survey flow

H1: Automatically enrolling adults for booster vaccine appointments (nudge) will increase (a) vaccination intentions and (b) policy support compared to leaving it up to adults to make their own appointments and not prompting them to reflect on the policy (control).

H2: Automatically enrolling adults for booster vaccine appointments and facilitating reflection on government's actions to promote booster uptake

(nudge+) will increase (a) vaccination intentions and (b) policy support compared to leaving it up to people to make their own appointments and not prompting them to reflect on the policy (control).

In particular, the nudge+ extends the nudge by encouraging people to think slowly about it. Banerjee and John [4] theorise that a nudge+ will increase the effectiveness of a nudge while also making it more acceptable because it respects individual autonomy. Based on early empirical evidence on transparent nudging [20, 7, 2], we therefore hypothesised the following:

H3: Automatically enrolling adults for booster vaccine appointments and facilitating reflection on government's actions to promote booster uptake (nudge+) will increase (a) vaccination intentions and (b) policy support compared to only automatically scheduling vaccine appointments for people (nudge).

All our hypotheses were preregistered on and are available via Open Science Framework.

Power analysis

Our sampling requirements were informed by a power analysis to detect an effect size of Cohen's d=0.05. This preregistered analysis was pooled across countries. Exploratory tests at the country level would test a total of 10 hypotheses in 7 countries across two different between-subjects experiments. Consequently, we used a conservative ex-ante Bonferroni multiple hypothesis correction which returned a type-I error of 0.0007 (=(α=0.05)/70. Using a t-test for two group independent means, our a priori total sample size requirement was N=20,238 (n=10,119) (one arm) with power of 0.95. The critical t associated with this analysis was 3.39 and the non-centrality parameter was 5.035. These sample size calculations were carried out using G*Power 3.1. Figure S2 shows our sampling distributions.

Descriptive statistics

Our sample is representative by age, education, gender, and subnational regions. On average, respondents are middle-aged (mean 47); those above 65 years of age make up the modal age category. The sample is fairly balanced by gender (male=48.3%) with nearly half of all respondents living either in large cities or suburbs. Just above a quarter of the sample is university-educated (at least a

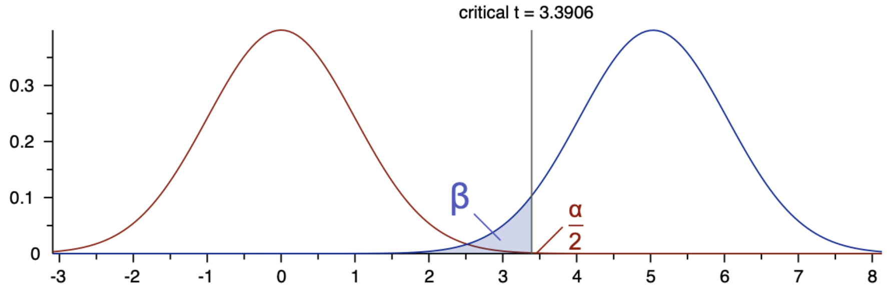

Figure S2: Sampling distribution

college graduate) (29.6%) and more than two-thirds of respondents have left-leaning political preferences (64.2%). Further, 22% of the sample do not trust COVID-19 vaccines and approximately 13% of the sample are totally unvaccinated. Vaccine hesitancy is significantly and negatively correlated with trust. Nonetheless, vaccine hesitancy significantly differs across the G7 countries: respondents in the US are most vaccine hesitant (26.3%) while those in Italy are least (9.5%).

Randomization checks

Following our preregistered analysis plan, we tested for balance of means in age (above 45 years of age or not), education (university educated or not), gender (male or female) and political ideology (left of center or not) across our four different experimental conditions. We do this by regressing each of these variables on our treatment dummies (nudge, think, nudge+) and testing the joint null hypothesis that all coefficients are zero. We find no evidence of imbalance by age (F=0.19, p=0.9036), education (F=2.04, p=0.1058), gender (F=0.65, p=0.5825), or political ideology (F=0.18, p=0.9100).

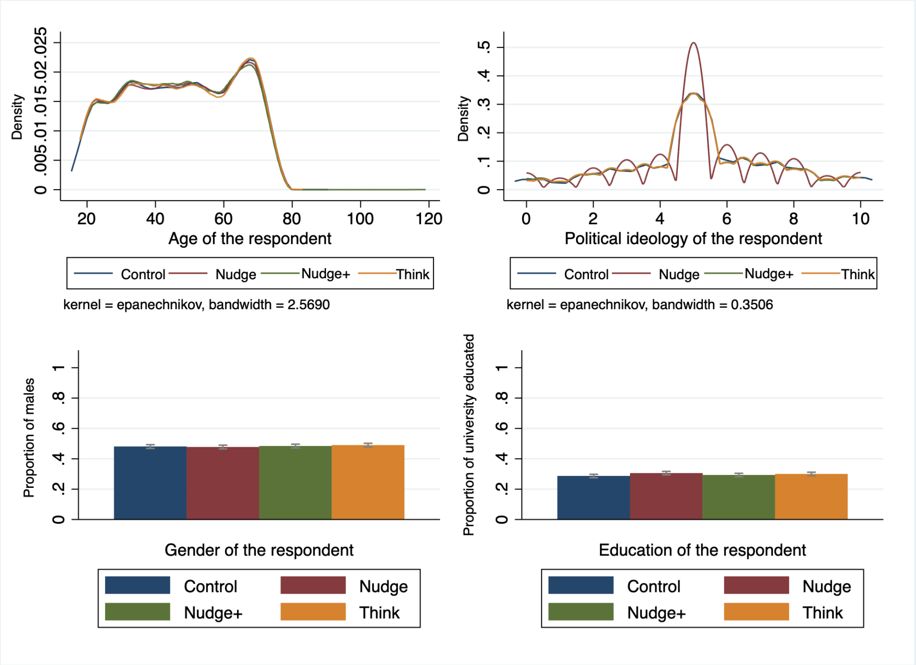

Figure S3: Density plots of demographic variables across experimental conditions

Country	Nudge	Think	Nudge+
UK	0.89	0.50	0.95
USA	0.29	0.97	0.67
Canada	0.35	0.05	0.23
France	0.43	0.45	0.92
Germany	0.06	0.31	0.01
Italy	0.24	0.27	0.56
Japan	0.39	0.72	0.65

Table S1: p-values corresponding to joint orthogonality test of balance of means of preregistered covariates across treatment conditions versus control

Demographics

Table S2: Distribution of individuals by age across G7 countries

G7	N	Mean	SD	Min	Max
UK	3472	46.674	16.568	18	75
USA	3483	45.747	16.421	18	88
Canada	3470	45.537	15.66	18	75
France	3485	47.192	16.389	18	75
Germany	3485	48.815	16.236	18	74
Italy	3487	48.352	16.081	18	119
Japan	3421	48.701	16.671	18	83

Table S3: Distribution of individuals by gender across G7 countries

	UK	USA	Canada	France	Germany	Italy	Japan	Total
A man	1682	1672	1720	1656	1682	1670	1652	11734
A woman	1768	1781	1722	1822	1796	1810	1755	12454
Another gender (please specify)	2	6	4	0	4	0	2	18
Non-binary	17	20	17	6	3	6	8	77
Prefer not to answer	3	4	7	1	0	1	4	20
Total	3472	3483	3470	3485	3485	3487	3421	24303

Table S4: Distribution of individuals by education across G7 countries

Country	College graduate		
	No	Yes	Total
UK	2875	597	3472
USA	2106	1377	3483
Canada	2567	903	3470
France	1846	1639	3485
Germany	2685	800	3485
Italy	2857	630	3487
Japan	2167	1254	3421
Total	17103	7200	24303

Table S5: Distribution of individuals by subnational regions in UK

UK regions	Freq.	Percent	Cum.
East Anglia	325	9.36	9.36
East Midlands	253	7.29	16.65
London	445	12.82	29.46
North East	147	4.23	33.7
North West	391	11.26	44.96
Northern Ireland	85	2.45	47.41
Prefer not to answer	3	0.09	47.49
Scotland	278	8.01	55.5
South East	476	13.71	69.21
South West	300	8.64	77.85
Wales	170	4.9	82.75
West Midlands	309	8.9	91.65
Yorkshire and Humberside	290	8.35	100
Total	3472	100	-

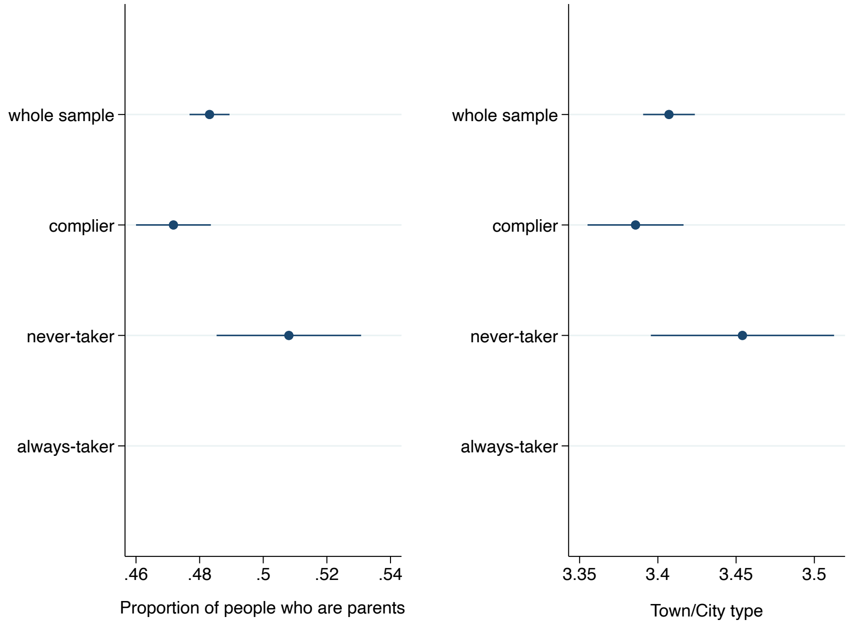

Figure S4: Complier profiling plots for nudge+ treatment

Table S6: Distribution of individuals by subnational regions in USA

USA regions	Freq.	Percent	Cum.
Alabama	39	1.12	1.12
Alaska	9	0.26	1.38
Arizona	97	2.78	4.16
Arkansas	34	0.98	5.14
California	363	10.42	15.56
Colorado	52	1.49	17.05
Connecticut	36	1.03	18.09
Delaware	18	0.52	18.60
District of Columbia	2	0.06	18.66
Florida	267	7.67	26.33
Georgia	125	3.59	29.92
Hawaii	24	0.69	30.61
Idaho	19	0.55	31.15
Illinois	124	3.56	34.71
Indiana	76	2.18	36.89
Iowa	31	0.89	37.78
Kansas	31	0.89	38.67
Kentucky	56	1.61	40.28
Louisiana	41	1.18	41.46
Maine	14	0.40	41.86
Maryland	49	1.41	43.27
Massachusetts	60	1.72	44.99
Michigan	117	3.36	48.35
Minnesota	54	1.55	49.90
Mississippi	23	0.66	50.56
Missouri	83	2.38	52.94
Montana	12	0.34	53.29
Nebraska	19	0.55	53.83
Nevada	56	1.61	55.44
New Hampshire	14	0.40	55.84
New Jersey	95	2.73	58.57
New Mexico	19	0.55	59.12
New York	214	6.14	65.26
North Carolina	122	3.50	68.76
North Dakota	3	0.09	68.85
Ohio	144	4.13	72.98
Oklahoma	40	1.15	74.13
Oregon	46	1.32	75.45
Pennsylvania	180	5.17	80.62
Prefer not to answer	1	0.03	80.65
Rhode Island	12	0.34	80.99
South Carolina	62	1.78	82.77
South Dakota	6	0.17	82.95
Tennessee	81	2.33	85.27
Texas	212	6.09	91.36
Utah	30	0.86	92.22
Vermont	6	0.17	92.39
Virginia	99	2.84	95.23
Washington	79	2.27	97.50
West Virginia	17	0.49	97.99
Wisconsin	62	1.78	99.77
Wyoming	8	0.23	100.00
Total	3483	100.00	-

Table S7: Distribution of individuals by subnational regions in Canada

Canadian regions	Freq.	Percent	Cum.
Alberta	439	12.65	12.65
British Columbia	371	10.69	23.34
Manitoba	182	5.24	28.59
New Brunswick	71	2.05	30.63
Newfoundland and Labrador	49	1.41	32.05
Nova Scotia	99	2.85	34.90
Nunavut	1	0.03	34.93
Ontario	1311	37.78	72.71
Prefer not to answer	1	0.03	72.74
Prince Edward Island	19	0.55	73.29
Quebec	810	23.34	96.63
Saskatchewan	115	3.31	99.94
Yukon	2	0.06	100.00
Total	3470	100.00	

Table S8: Distribution of individuals by subnational regions in France

French regions	Freq.	Percent	Cum.
Alsace	84	2.41	2.41
Aquitaine	191	5.48	7.89
Auvergne	76	2.18	10.07
Basse-Normandie	57	1.64	11.71
Bourgogne	104	2.98	14.69
Bretagne	189	5.42	20.11
Centre	152	4.36	24.48
Champagne-Ardenne	84	2.41	26.89
Corse	14	0.40	27.29
Franche-Comte	59	1.69	28.98
Haute-Normandie	100	2.87	31.85
Languedoc-Roussillon	152	4.36	36.21
Limousin	33	0.95	37.16
Lorraine	169	4.85	42.01
Midi-Pyrences	161	4.62	46.63
Nord-Pas-de-Calais	208	5.97	52.60
Pays de la Loire	217	6.23	58.82
Picardie	98	2.81	61.64
Poitou-Charentes	83	2.38	64.02
Prefer not to answer	4	0.11	64.13
Provence-Alpes-Cyte d'Azur	280	8.03	72.17
Rhyne-Alpes	343	9.84	82.01
éle-de-France	627	17.99	100.00
Total	3485	100.00	-

Table S9: Distribution of individuals by subnational regions in Germany

German regions	Freq.	Percent	Cum.
ABaden-Worttemberg	455	13.06	13.06
Bayern	538	15.44	28.49
Berlin	149	4.28	32.77
Brandenburg	103	2.96	35.72
Bremen	23	0.66	36.38
Hamburg	99	2.84	39.23
Hessen	275	7.89	47.12
Mecklenburg-Vorpommern	78	2.24	49.35
Niedersachsen	319	9.15	58.51
Nordrhein-Westfalen	758	21.75	80.26
Rheinland-Pfalz	163	4.68	84.94
Saarland	41	1.18	86.11
Sachsen	191	5.48	91.59
Sachsen-Anhalt	100	2.87	94.46
Schleswig-Holstein	114	3.27	97.73
Thoringen	79	2.27	100.00
Total	3485	100.00	-

Table S10: Distribution of individuals by subnational regions in Italy

Italian regions	Freq.	Percent	Cum.
Abruzzo	82	2.35	2.35
Basilicata	33	0.95	3.30
Calabria	109	3.13	6.42
Campania	339	9.72	16.15
Emilia-Romagna	270	7.74	23.89
Friuli-Venezia Giulia	82	2.35	26.24
Lazio	338	9.69	35.93
Liguria	92	2.64	38.57
Lombardia	566	16.23	54.80
Marche	89	2.55	57.36
Molise	20	0.57	57.93
Piemonte	259	7.43	65.36
Puglia	235	6.74	72.10
Sardegna	101	2.90	74.99
Sicilia	295	8.46	83.45
Toscana	211	6.05	89.50
Trentino-Alto Adige	40	1.15	90.65
Umbria	47	1.35	92.00
Valle d'Aosta	4	0.11	92.11
Veneto	275	7.89	100.00
Total	3487	100.00	-

Table S11: Distribution of individuals by subnational regions in Japan

Japanese regions	Freq.	Percent	Cum.
Aichi	198	5.79	5.79
Akita	26	0.76	6.55
Aomori	39	1.14	7.69
Chiba	163	4.76	12.45
Ehime	46	1.34	13.80
Fukui	20	0.58	14.38
Fukuoka	159	4.65	19.03
Fukushima	39	1.14	20.17
Gifu	52	1.52	21.69
Gunma	36	1.05	22.74
Hiroshima	95	2.78	25.52
Hokkaido	152	4.44	29.96
Hyogo	162	4.74	34.70
Ibaraki	62	1.81	36.51
Ishikawa	28	0.82	37.33
Iwate	32	0.94	38.26
Kagawa	32	0.94	39.20
Kagoshima	34	0.99	40.19
Kanagawa	247	7.22	47.41
Kochi	12	0.35	47.76
Kumamoto	44	1.29	49.05
Kyoto	63	1.84	50.89
Mie	43	1.26	52.15
Miyagi	81	2.37	54.52
Miyazaki	29	0.85	55.36
Nagano	43	1.26	56.62
Nagasaki	32	0.94	57.56
Nara	38	1.11	58.67
Niigata	68	1.99	60.65
Oita	30	0.88	61.53
Okayama	34	0.99	62.53
Okinawa	25	0.73	63.26
Osaka	265	7.75	71.00
Prefer not to answer	3	0.09	71.09
Saga	23	0.67	71.76
Saitama	189	5.52	77.29
Shiga	28	0.82	78.11
Shimane	14	0.41	78.52
Shizuoka	87	2.54	81.06
Tochigi	52	1.52	82.58
Tokushima	15	0.44	83.02
Tokyo	426	12.45	95.47
Tottori	19	0.56	96.02
Toyama	20	0.58	96.61
Wakayama	26	0.76	97.37
Yamagata	29	0.85	98.22
Yamaguchi	38	1.11	99.33
Yamanashi	23	0.67	100.00
Total	3421	100.00	-

Table S12: Start and end dates of survey in G7 countries

Country	Start date	End date
UK	27-Jan-22	21-Feb-22
USA	27-Jan-22	26-Feb-22
Canada	27-Jan-22	24-Feb-22
France	27-Jan-22	17-Feb-22
German	27-Jan-22	11-Feb-22
Italy	27-Jan-22	08-Feb-22
Japan	27-Jan-22	11-Feb-22

Table S13: Summary statistics of outcome variables

	Treatment	N	Mean	S.D.
Intentions	Control	6,166	4.703	1.679
	Nudge	6,057	4.668	1.714
	Nudge+	5,947	4.607	1.778
	Think	6,042	4.688	1.689
	All	24,212	4.669	1.715
Approval	Control	6,140	6.327	2.881
	Nudge	6,045	6.311	3.077
	Nudge+	5,957	6.195	3.197
	Think	6,026	6.411	2.801
	All	24,168	6.311	2.993

Table S14: Intent to treat effects on vaccination intentions with randomized missingness in nudge+ condition

	(1)	(2)	(3)	(4)	(5)	(6)
Nudge	-0.065***	-0.065***	-0.065***	-0.065***	-0.065***	-0.065***
	(0.021)	(0.021)	(0.021)	(0.021)	(0.021)	(0.021)
Think	-0.058***	-0.058***	-0.058***	-0.058***	-0.058***	-0.058***
	(0.021)	(0.021)	(0.021)	(0.021)	(0.021)	(0.021)
Nudge+	-0.125***	-0.122***	-0.123***	-0.123***	-0.123***	-0.117***
	(0.021)	(0.021)	(0.021)	(0.021)	(0.021)	(0.021)
Controls	✓	✓	✓	✓	✓	✓
Country FE	✓	✓	✓	✓	✓	✓
Nudge − nudge+	0.059***	0.056**	0.058**	0.057**	0.058**	0.052*
	(0.020)	(0.021)	(0.021)	(0.021)	(0.021)	(0.021)
N	24,164	24,105	24,045	23,986	23,930	23,866

OLS estimates with robust standard errors in parentheses; *** p<0.005, ** p<0.01, * p<0.05. Controls selected by lasso linear regression specification. All models include controls for age, gender, parental status, town/city type, religious beliefs, prior COVID–19 infection status (self), vaccination status, booster status, and trust in vaccines (binary). Column 1 retains all nudge+ observations. Columns 2–6 retain 95–99% nudge+ observations by randomly dropping 1% of observations in each column progressively.

Table S15: Intent to treat effects on policy approval with randomized missingness in nudge+ condition

	(1)	(2)	(3)	(4)	(5)	(6)
Nudge	-0.037	-0.037	-0.037	-0.037	-0.037	-0.037
	(0.048)	(0.048)	(0.048)	(0.048)	(0.048)	(0.048)
Think	0.035	0.035	0.035	0.035	0.035	0.035
	(0.048)	(0.048)	(0.048)	(0.048)	(0.048)	(0.048)
Nudge+	-0.149***	-0.149***	-0.142***	-0.148***	-0.138***	-0.150***
	(0.048)	(0.048)	(0.048)	(0.048)	(0.048)	(0.048)
Controls	✓	✓	✓	✓	✓	✓
Country FE	✓	✓	✓	✓	✓	✓
Nudge − nudge+	0.112*	0.112*	0.105*	0.112*	0.101*	0.113*
	(0.048)	(0.048)	(0.048)	(0.048)	(0.049)	(0.049)
N	24,115	24,055	23,997	23,937	23,878	23,818

OLS estimates with robust standard errors in parentheses; *** p<0.005, ** p<0.01, * p<0.05. Controls selected by lasso linear regression specification. All models include controls for age, gender, parental status, religious beliefs, prior COVID–19 infection status (self), booster status, and trust in vaccines (binary). Column 1 retains all nudge+ observations. Columns 2–6 retain 95–99% nudge+ observations by randomly dropping 1% of observations in each column progressively.

Table S16: Complier average causal effects for treatment conditions

	Intentions (1)	Approval (2)	Intentions (3)	Approval (4)	Intentions (5)	Approval (6)
Nudge	-0.094***	-0.054	-0.096***	-0.055	-0.094***	-0.054
	(0.029)	(0.066)	(0.029)	(0.067)	(0.029)	(0.066)
Think	-0.063***	0.038	-0.506***	0.293	-0.241***	0.145
	(0.022)	(0.053)	(0.173)	(0.422)	(0.083)	(0.203)
Nudge+	-0.108***	-0.129***	-1.196***	-1.429***	-0.458***	-0.548***
	(0.018)	(0.041)	(0.201)	(0.458)	(0.077)	(0.175)
Controls	✓	✓	✓	✓	✓	✓
Country FE	✓	✓	✓	✓	✓	✓
Instrument	Sentences written		Sentences (standardised)		$\sqrt{\# \text{characters}}$ (standardised)	
Nudge+ − nudge	-0.013	-0.074	-1.100***	-1.347***	-0.363***	-0.494***
	(0.026)	(0.058)	(0.189)	(0.428)	(0.069)	(0.153)
Nudge+ − think	-0.045*	-0.166***	-0.689***	-1.722***	-0.217**	-0.693***
	(0.021)	(0.049)	(0.196)	(0.452)	(0.084)	(0.196)
N	24164	24115	24164	24115	24164	24115

Two-stage least squares estimates with robust standard errors in parentheses; *** p<0.005, ** p<0.01, * p<0.05 (Young 51 randomization-*t* *p*-values for mean differences of experimental conditions versus control condition). We instrument for measures of nudge, think, and nudge+ treatment compliance using indicators for each experimental condition. Columns 1–2 instrument nudge+ and think using number of sentences written conditional on answering the manipulation check correctly. Columns 3–4 instrument nudge+ and think using number of sentences written standardised by country conditional on answering the manipulation check correctly. Columns 5–6 instrument nudge+ and think using the square root of total characters written by each respondent standardised by country conditional on answering manipulation check correctly. Controls selected by lasso linear regression specification as in Table 3.

Table S17: Intent to treat effects on vaccination intentions by country

	UK	US	Canada	France	Germany	Italy	Japan
Nudge	0.001	-0.252***	-0.046	0.033	-0.182***	0.001	-0.030
	(0.049)	(0.058)	(0.055)	(0.059)	(0.050)	(0.048)	(0.050)
Think	-0.097*	-0.067	-0.057	0.051	-0.094	0.054	-0.179***
	(0.048)	(0.056)	(0.054)	(0.058)	(0.052)	(0.046)	(0.052)
Nudge+	-0.109*	-0.241***	-0.121*	-0.029	-0.235***	0.009	-0.127**
	(0.051)	(0.057)	(0.0.57)	(0.060)	(0.053)	(0.048)	(0.056)
Controls	✓	✓	✓	✓	✓	✓	✓
Nudge+ − Nudge	-0.109*	0.010	-0.075	-0.063	-0.052	0.008	-0.097
	(0.052)	(0.061)	(0.058)	(0.061)	(0.054)	(0.0499)	(0.056)
N	3462	3466	3406	3471	3478	3481	3400

OLS estimates with robust standard errors in parentheses; *** p<0.005, ** p<0.01, * p<0.05 (Young 51 randomization-*t* *p*-values for mean differences of experimental conditions versus control condition). Controls selected by lasso linear regression specification as in Table 3.

Table S18: Intent to treat effects on policy approval by country

	UK	US	Canada	France	Germany	Italy	Japan
Nudge	0.440***	-0.606***	-0.093	-0.198	-0.123	0.039	0.223*
	(0.117)	(0.138)	(0.128)	(0.121)	(0.121)	(0.116)	(0.103)
Think	-0.298*	0.519***	0.043	0.354***	-0.137	-0.337**	0.117
	(0.127)	(0.144)	(0.132)	(0.125)	(0.136)	(0.129)	(0.109)
Nudge+	0.328*	-0.596***	-0.380***	-0.282*	-0.418**	0.123	0.182
	(0.124)	(0.140)	(0.135)	(0.124)0	(0.128)	(0.117)	(0.109)
Controls	✓	✓	✓	✓	✓	✓	✓
Nudge+ − Nudge	-0.111	0.010	-0.287*	-0.083	-0.295*	0.084	-0.041
	(0.115)	(0.139)	(0.133)	(0.126)	(0.125)	(0.110)	(0.107)
N	3459	3463	3403	3468	3475	3473	3374

OLS estimates with robust standard errors in parentheses; *** p<0.005, ** p<0.01, * p<0.05 (Young 51 randomization-*t* *p*-values for mean differences of experimental conditions versus control condition). Controls selected by lasso linear regression specification as in Table 3.

Table S19: Intent to treat effects estimated as a 2×2 factorial design

	Intentions	Approval
Default	-0.065***	-0.037
	(0.020)	(0.046)
Reflection	-0.058***	0.035
	(0.020)	(0.049)
Default × Reflection	-0.001	-0.147*
	(0.029)	(0.068)
Controls	✓	✓
Country FE	✓	✓
N	24164	24115

OLS estimates with robust standard errors in parentheses; *** p<0.005, ** p<0.01, * p<0.05 (Young 51 randomization-*t* *p*-values). Controls selected by lasso linear regression specification. Column 1 includes controls for age, gender, parental status, town/city type, religious beliefs, prior COVID–19 infection status (self), vaccination status, booster status, and trust in vaccines (binary). Column 1 retains all nudge+ observations. Column 2 includes controls for age, gender, parental status, religious beliefs, prior COVID–19 infection status (self), booster status, and trust in vaccines (binary).

Table S20: Character transformation ratio by country

Language	Words in survey preamble	Minimum characters in open-text
English	1322	75
French	1821	100
Italian	1576	90
German	1630	90
Japanese	625	35

Heterogeneity analysis

We estimate heterogeneous effects of nudge, nudge+ and think on vaccination intentions and policy support (relative to the control condition) using a causal forest model. To ensure comparability between outcomes, we rescale both dependent variables to a range of 0–1. Comparisons between this analysis and the main analysis can be facilitated by dividing effect sizes in the main analysis by 5 for intentions and by 11 for support. Figure S5 shows the distribution of predicted individual-level treatment effects for both intentions and support.

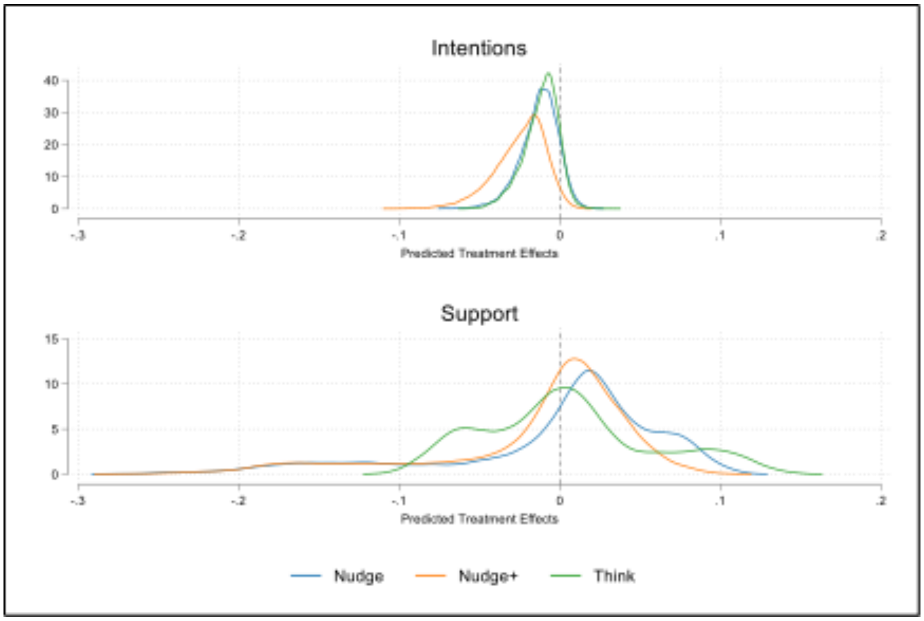

Figure S5: Estimated treatment effects from causal forest model

The predicted treatment effects of all three conditions on intentions are overwhelmingly negative. Indeed, exposure to the nudge condition is expected to produce a negative effect in 88% of respondents, while exposure to the nudge+ condition is expected to do so for 97% and exposure to the think condition is expected to produce a negative effect in 89%. For the nudge and think conditions, the modal outcome is approximately -0.01 which, accounting for rescaling, is consistent with the results of the main analysis. Similarly, the high concentration of observations around this value shows that there is little heterogeneity associated with either of these treatments. For the nudge+ treatment, we see evidence of modest heterogeneity (the thicker tail on the left side of the dis-

tribution) but the average predicted treatment effect of the nudge+ treatment on vaccine intentions is 0.025, which is again consistent with the results of the main analysis after accounting for rescaling.

When we turn to support, we see substantially more variance in predicted treatment effects. Indeed, the nudge condition is expected to produce a negative effect in 35% of respondents, while the nudge+ condition is expected to do so in 44% of respondents and the think condition is expected to do so in 50% of respondents. Accounting for rescaling, the average predicted treatment effect for each condition is consistent with the results of the main analysis: -0.003 for the nudge condition, -0.015 for the nudge+ condition, and 0.004 for the think condition. Furthermore, there is considerable heterogeneity in estimated treatment effects across all conditions, with a range of 0.42 in the nudge condition, 0.41 in the nudge+ condition, and 0.28 in the think condition (all of which are substantive given the 0-1 rescaling). Indeed, the modal outcome of each condition are in fact positive, meaning the negative and statistically significant result found with respect to the nudge+ condition is driven in large part by those respondents with a strong negative reaction to the treatment.

Table S21 shows the linear effect of each covariate on the estimated treatment effect associated with nudge+ on intentions and support, respectively. Unsurprisingly, the impact of each covariate is similar across models. Of the covariates for which p¡0.10 for intentions, we see that men and participants without a booster, who are less trusting in institutions, and who are more right-leaning are less likely to have a positive reaction to nudge+. We also see that nudge+ was most effective in Japan (the reference category for country estimates) as evidenced by the negative and statistically significant coefficients associated with the other six countries.

Variable	nudge+ (intentions)	nudge+ (approval)
Age (1–6)	-.002	.014
	(.007)	(.014)
Male (0–1)	-.032†	-.022
	(.017)	(.034)
College education (0–1)	.013	.026
	(.018)	(.039)
Urban-rural (1–5)	.005	-.005
	(.006)	(.014)
Religion (1–4)	.004	-.015
	(.008)	(.017)
Full-time worker (0–1)	.033	.022
	(.055)	(.097)
Part-time worker (0–1)	.057	.036
	(.055)	(.099)
Self-employed (0–1)	.033	.079
	(.06)	(.106)
Retired (0–1)	.049	-.062
	(.061)	(.116)
Unemployed (0–1)	.021	-.118
	(.063)	(.11)
Student (0–1)	.065	-.013
	(.058)	(.107)
Caring (0–1)	.069	.002
	(.063)	(.122)
Parent (0–1)	-.011	-.028
	(.017)	(.035)
No dose (0–1)	.008	-.065
	(.039)	(.073)
Partial dose (0–1)	-.026	.05
	(.035)	(.061)
No booster (0–1)	-.084***	-.13**
	(.026)	(.048)
Family member had COVID (0–1)	-.045*	-.039
	(.019)	(.04)
General trust in vaccines (0–1)	.089*	.064
	(.038)	(.065)
COVID vaccine attitudes (standardised)	0	.006
	(.016)	(.032)
COVID news, newspaper (0–1)	.024	-.128
	(.052)	(.098)
COVID news, Radio (0–1)	.015	-.043
	(.058)	(.112)
COVID news, social media (0–1)	.018	-.015
	(.053)	(.097)
COVID news, TV (0–1)	.027	-.107
	(.05)	(.092)
COVID news, WeChat (0–1)	.111	.289†
	(.105)	(.152)
COVID news volume (1–6)	.009	.024†
	(.007)	(.014)
Lives-liberty tradeoff (0–10)	.005	.025**
	(.004)	(.009)
COVID actions (standardised)	.016	.044*
	(.01)	(.022)
Trust in institutions (standardised)	.028**	.068**
	(.01)	(.023)
Populism (standardised)	.001	-.01
	(.01)	(.021)
Left-right (0–10)	-.011**	-.013†
	(.004)	(.008)
Risk preferences (0–10)	-.003	0
	(.004)	(.008)
UK (0–1)	-.09*	-.038
	(.038)	(.07)
US (0–1)	-.089*	-.197**
	(.039)	(.074)
Canada (0–1)	-.142***	-.3***
	(.037)	(.071)
France (0–1)	-.083*	-.281***
	(.039)	(.071)
Germany (0–1)	-.16***	-.435***
	(.036)	(.071)
Italy (0–1)	-.093**	-.136*
	(.036)	(.066)
(Constant)	.053	.129
	(.1)	(.184)

Table S21: Heterogeneity in treatment effects by covariates in nudge+ condition; *** p<0.001, ** p<0.01, * p<0.05

Meanwhile, with respect to approval and again using a $p < 0.1$ criterion, we see those without a booster, those less trusting in institutions and those more right-leaning are more likely to exhibit a negative reaction to the nudge+ treatment. Conversely, those who use WeChat to access COVID news, those who consume more COVID news, those who prioritise the saving of lives over personal liberty and those who are more compliant with social distancing and mask mandates are more likely to exhibit a positive reaction to the nudge+ treatment.

Text analysis

Respondents in the nudge+ and think conditions were invited to share their thoughts on the policies to which they were exposed via a text entry box. To analyse these responses, we first translate the non-English language responses to English using Google Translate. We then count the frequency of each word using the Natural Language Toolkit (nltk) package in Python, excluding commonly used but low information words (e.g., "and", "are", "any", etc.) using nltk's "stop words" list. We calculate the frequency of words per experimental condition in both absolute (i.e., the raw frequency of each word in each condition) and relative (i.e., the difference in frequency for each word between conditions).

	**Absolute frequency**		**Relative frequency**	
	Nudge+	Think	Nudge+	Think
1	think (1759)	think (1696)	good (+234)	booster (+245)
2	would (1406)	people (1302)	automatically (+137)	people (+205)
3	people (1097)	would (1283)	opt (+132)	choice (+191)
4	get (913)	get (1031)	would (+123)	everyone (+175)
5	appropriate (808)	appropriate (777)	automatic (+122)	make (+175)
6	good (768)	government (771)	convenient (+119)	individual (+165)
7	yes (694)	vaccine (732)	idea (+117)	vaccination (+153)
8	government (692)	everyone (732)	registration (+97)	decide (+141)
9	vaccine (636)	vaccinated (714)	refuse (+94)	whether (+139)
10	approach (629)	yes (681)	time (+83)	none (+125)

Table S22: Word Frequency across think and nudge+ conditions

Table S22 presents the frequency of the 10 most common words used by respondents per experimental condition in both absolute and relative terms. Perhaps unsurprisingly, in absolute terms, the most frequently used words differ little between conditions and pertain mostly to the domain of vaccine policy and the appropriateness thereof. By contrast, the relative frequencies are more revealing of differences in respondents' perceptions of the two supposed policies. In

the case of nudge+, respondents appear to focus more on the ease by this policy would facilitate vaccination with words like "automatic" and "convenient" ranking among those with the highest relative frequency. There is evidence of a positive reaction to this policy among many respondents with "good" being the most frequently used word in this condition (relative to the think condition). However, there is also evidence of a negative reaction amongst some respondents with the word "refuse" also appearing in the top 10 of the relative frequency. Meanwhile, respondents in the think condition appear to focus more on the extent to which this policy preserves individual agency with words like "choice" and "decide" appearing more often. A more extensive representation of the top 200 words per experimental condition is presented in Figure S6 in the form of a word cloud where the size of each word is proportionalto the frequency of the word.

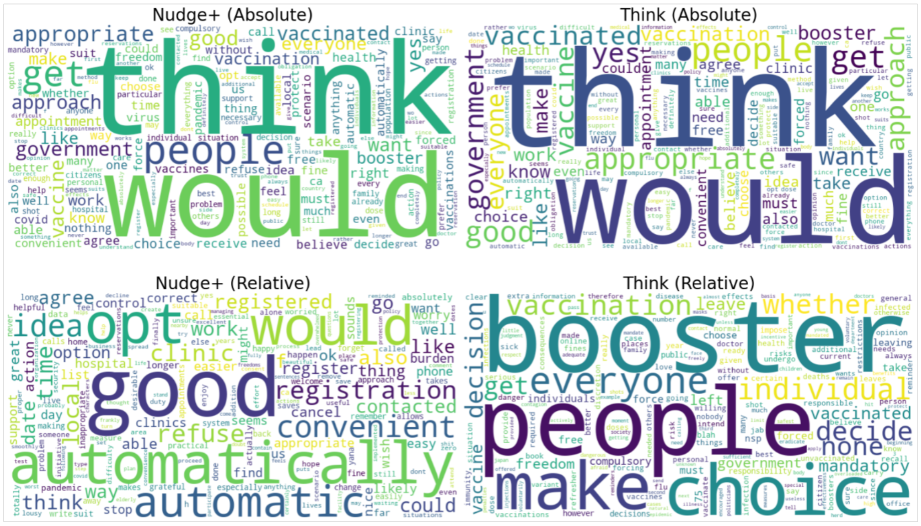

Figure S6: Word cloud illustrating word frequency in responses regarding respondent vaccine policy reflections (excluding low content words). Word size reflects word frequency in the nudge+ and think conditions, respectively. Word frequency is displayed in both absolute (i.e., per each condition, independent of the other) and relative terms (i.e., frequency difference between conditions). 200 words per panel.

We also examined a randomly selected subset of 150 responses to further identify the key themes underlying attitudes towards these policies. First, respondents (particularly those in the nudge+ condition) focused on the ease of receiving a

vaccine, noting that automatic enrolment would make it easier for them (and likely others) to make an appointment and reducing the possibility of forgetting to make an appointment. For example, one respondent commented "I think this approach is appropriate and I would find it very convenient if someone called me to schedule an appointment.". Conversely, a number of respondents in the think condition noted the reverse — i.e., that many respondents would fail to make an appointment if individuals had to choose to opt-in. For instance, another respondent commented "i think a lot of people just won[']t bother if they have to sort it out themselves."

Second, a number of respondents in both conditions also noted their perception that the policies were detrimental to individual agency, though this reaction was most notable in the nudge+ group. Participants who perceived nudge+ as detrimental to individual agency tended to misperceived the policy as mandatory vaccination ("I think it would be better to be able to refuse automatic registration instead of forcing people to be vaccinated"), an objection to the policy as undermining individual agency ("I think there's too much coercion behind it. There will be citizens who feel responsible just because the date was set by the government..."), practical concerns over the timing and location of the appointment ("I prefer to do my booster dose with my GP and not in a clinic further away from where I live."), or some combination of the three. Meanwhile, respondents in the think group who mentioned individual agency tended to do so on the basis of practical concerns ("No do not contact a clinic, go through a doctor or pharmacist or health professional").

Third, a roughly equal proportion of respondents in nudge+ and think groups noted the appropriateness of the retention of individual agency associated with either policy. For example, one respondent in the nudge+ condition commented, "This approach would suit me perfectly. We still have the option to withdraw," while another in the think condition commented, "I agree that adults should be able to choose. I don't mind calling to schedule".

Fourth, a number of respondents commented on the appropriateness of either policy in light of its expected positive effect on public health and the lifting of pandemic restrictions, again this was roughly equal proportion across groups — for example, "These policies are sufficient to bring the pandemic under control, and personally, I would like to get vaccinated."

Survey questionnaire

The U.K. questionnaire is provided below. All questionnaires are available online at https://www.dropbox.com/sh/3zud2xefz1lyvet/AACrGdHvj9yZ8FA1jzYFQ5eFa?dl=0.

UK

Survey Flow

EmbeddedData
psidValue will be set from Panel or URL.

Standard: ethics (2 Questions)

Branch: New Branch
If
If Thank you for taking the time to participate in this study.   Please note that you need to be 18... I DO NOT give my consent to participate in this research study. Is Selected

EndSurvey: Advanced

Branch: New Branch
If
If What is your age? Please enter as a number (e.g., 25). Text Response Is Less Than or Equal to 17

EndSurvey: Advanced

Standard: captcha (1 Question)
Standard: Screens1 (1 Question)

Branch: New Branch
If
If Help us keep track of who is paying attention - please select “somewhat disagree” in the options... Somewhat disagree Is Not Selected

EndSurvey: Advanced

Standard: Demographics (13 Questions)

Branch: New Branch
If
If Do you currently live in the United Kingdom? No Is Selected

EndSurvey: Advanced

Standard: Party ID (3 Questions)
Standard: Scales (5 Questions)
Standard: covid19 (16 Questions)
Standard: Media consumption (2 Questions)
Standard: Screen2 (1 Question)
Standard: conjoint (19 Questions)
Standard: screens3 (1 Question)

BlockRandomizer: 1 -

Standard: treatment1 (9 Questions)

Standard: treatment2 (9 Questions)
Standard: treatment3 (10 Questions)
Standard: treatment4 (10 Questions)

Standard: post-treatment (8 Questions)

EmbeddedData
effectiveValue will be set from Panel or URL.
scheduleValue will be set from Panel or URL.
remindersValue will be set from Panel or URL.
mandateValue will be set from Panel or URL.
finesValue will be set from Panel or URL.
schedule1Value will be set from Panel or URL.
reminders1Value will be set from Panel or URL.
mandate1Value will be set from Panel or URL.
fines1Value will be set from Panel or URL.
schedule2Value will be set from Panel or URL.
reminders2Value will be set from Panel or URL.
mandate2Value will be set from Panel or URL.
fines2Value will be set from Panel or URL.
schedule3Value will be set from Panel or URL.
reminders3Value will be set from Panel or URL.
mandate3Value will be set from Panel or URL.
fines3Value will be set from Panel or URL.
schedule4Value will be set from Panel or URL.
reminders4Value will be set from Panel or URL.
mandate4Value will be set from Panel or URL.
fines4Value will be set from Panel or URL.
schedule5Value will be set from Panel or URL.
reminders5Value will be set from Panel or URL.
mandate5Value will be set from Panel or URL.
fines5Value will be set from Panel or URL.
Q_TerminateFlagValue will be set from Panel or URL.

EndSurvey: Advanced

Page Break

Start of Block: ethics

JS

ethics <div> <p dir="ltr">Thank you for taking the time to participate in this study. </p> <p dir="ltr"> </p> <p dir="ltr">Please note that you need to be 18+ and speak English fluently, otherwise please don't partake. The study should take around 15 minutes to complete. In the study, you will be asked a series of questions about vaccines. You will be paid for your participation in the survey. Just make sure to read all the instructions carefully and try your best. </p> <p dir="ltr"> </p> <p dir="ltr">NOTE: You can withdraw from the study at any stage without providing an explanation. Your privacy is very important, so we always use anonymised data. Results from this work may be written up for publication in a peer reviewed journal. However, individual data will never be published, and we will not hold personal identifiers. This project is in line with the ethical guidelines established by the Research Ethics Committee of King's College London.</p> <p dir="ltr"> </p> For more details about this research project, please see this information sheet.   <div> </div> <div>If you have any questions you would like to ask before starting the survey, please feel free to contact Professor Peter John, King's College London: peter.john@kcl.ac.uk. </div> </div> <div> </div> <div>Please note in this survey we would like to ask some questions that may be perceived as sensitive, such as gender, ethnicity, political orientation, and religion. Providing information in response to these questions is entirely voluntary and you may withdraw your consent at any time. If you are happy to participate, please choose "I give my consent to participate in this research study."</div> <div> <div> </div> </div>

- ◯ I DO NOT give my consent to participate in this research study. (1)
- ◯ I give my consent to participate in this research study. (2)

Skip To: End of Block If ethics = 1

Page Break

*

yob What is your age? Please enter as a number (e.g., 25).

____

Skip To: End of Block If Condition: What is your age? Please en... Is Less Than or Equal to 17. Skip To: End of Block.

End of Block: ethics

Start of Block: captcha

captcha Before you proceed to the survey, please complete the Captcha below.

End of Block: captcha

Start of Block: Screens1

screen1 Help us keep track of who is paying attention - please select “somewhat disagree” in the options below.

- ○ Strongly disagree (1)
- ○ Somewhat disagree (2)
- ○ Neither agree nor disagree (3)
- ○ Somewhat agree (4)
- ○ Strongly agree (5)

Skip To: End of Block If screen1 = 1

Skip To: End of Block If screen1 = 3

Skip To: End of Block If screen1 = 4

Skip To: End of Block If screen1 = 5

End of Block: Screens1

Start of Block: Demographics

country Do you currently live in the United Kingdom?

- ◯ Yes (1)
- ◯ No (2)

Skip To: End of Block If country = 2

Page Break

subnat_region Which region do you currently live in?

- East Anglia (1)
- East Midlands (3)
- London (4)
- North East (5)
- North West (6)
- Northern Ireland (7)
- Scotland (8)
- South East (9)
- South West (10)
- Wales (11)
- West Midlands (12)
- Yorkshire & Humberside (13)
- Prefer not to answer (2)

Page Break

gender Are you...

- ○ A man (1)
- ○ A woman (2)
- ○ Non-binary (3)
- ○ Another gender (please specify) (4) ____
- ○ Prefer not to answer (5)

Page Break

education What is the highest level of education you have achieved?

○ Combined Junior and Infant School/ Infant School (1)

○ Junior School (11)

○ Comprehensive School (12)

○ Comprehensive School (GCSE)/ Secondary Modern (GCSE)/ Grammar School (GCSE)/ City Technology College (GCSE)/ Sixth Form (13)

○ College/ Institution of Higher education (14)

○ Open College - College of Technology - Institute/ Teacher Training College (15)

○ University/ Open University (16)

○ Prefer not to answer (2)

Page Break

citizen Are you a citizen of the United Kingdom?

- ○ Yes (1)
- ○ No (2)

Page Break

parent_screen Are you a parent or guardian to any children?

○ Yes (1)

○ No (2)

Page Break

Display This Question:

If parent_screen = 1

X→

children How many children under the age of 18 are you the parent or guardian of?

- 0 (4)
- 1 (25)
- 2 (5)
- 3 (6)
- 4 (7)
- 5 (8)
- 6 (9)
- 7 (10)
- 8 (11)
- 9 (12)
- 10 (13)
- 11 (14)
- 12 (15)
- 13 (16)
- 14 (17)
- 15 (18)
- 16 (19)
- 17 (20)
- 18 (21)
- 19 (22)
- 20 or more (24)

Page Break

children_u18 Are there any children under the age of 18 living in your household?

- 0 (1)
- 1 (2)
- 2 (3)
- 3 (4)
- 4 (5)
- 5 (6)
- 6 (7)
- 7 (8)
- 8 (9)
- 9 (10)
- 10 (11)
- 11 (12)
- 12 (13)
- 13 (14)
- 14 (15)
- 15 (16)
- 16 (17)
- 17 (18)
- 18 (19)
- 19 (20)
- 20 or more (21)

Page Break

ethnicity Which of the following best describes your ethnicity?

- [ ] White: British (1)
- [ ] White: Irish (2)
- [ ] White: Other (3)
- [ ] Mixed: White and Black Caribbean (4)
- [ ] Mixed: White and Black African (5)
- [ ] Mixed: White and Asian (6)
- [ ] Mixed: Other mixed background (7)
- [ ] Black or Black British: African (8)
- [ ] Black or Black British: Caribbean (9)
- [ ] Black or Black British: Any other Black background (10)
- [ ] Asian or Asian British: Indian (11)
- [ ] Asian or Asian British: Pakistani (12)
- [ ] Asian or Asian British: Bangladeshi (13)
- [ ] Asian or Asian British: Other Asian background (14)
- [ ] Chinese (15)
- [ ] Other ethnic group not represented by these options (please specify) (16) ____
- [ ] Do not wish to say (17)

Page Break

employment What is your employment status? Please select as many as applicable.

- ☐ Working for pay full-time (1)
- ☐ Working for pay part-time (2)
- ☐ Self-employed (3)
- ☐ Retired (4)
- ☐ Unemployed / Looking for work (5)
- ☐ Student (6)
- ☐ Caring for family (7)
- ☐ Other (Please specify) (8) ____

Page Break

income What was your total household income, before taxes, for the year 2021?

- No income (1)
- £1 - £4,400 (2)
- £4,401 - £8,800 (4)
- £8,801 - £17,600 (5)
- £17,601 - £26,400 (6)
- £26,401 - £35,200 (7)
- £35,201 - £52,800 (8)
- £52,801 - £64,500 (9)
- £64,501 - £88,000 (10)
- £88,001 - £117,300 (11)
- More than £117,300 (12)
- Don't know / prefer not to answer (3)

Page Break

urban_rural Which of the following best describes the place where you now live…

- ○ A large city (1)
- ○ A suburb near a large city (2)
- ○ A small city (3)
- ○ A town (4)
- ○ A rural area (5)

Page Break

religiosity In your life, you would say religion is:

- ○ Very important (1)
- ○ Somewhat important (2)
- ○ Not very important (3)
- ○ Not at all important (4)

End of Block: Demographics

Start of Block: Party ID

partyid In politics, do you usually think of yourself as a(n):

- ○ Conservative Party (1)
- ○ Labour Party (2)
- ○ Scottish National Party (3)
- ○ Liberal Democrats (4)
- ○ Democratic Unionist Party (5)
- ○ Sinn Féin (6)
- ○ Plaid Cymru (7)
- ○ Social Democratic and Labour Party (8)
- ○ Green Party (9)
- ○ Alliance Party of Northern Ireland (10)
- ○ Another party (specify): (11) ____
- ○ No party (12)
- ○ Don't know (13)

Page Break

Display This Question:

If partyid = 1

Or partyid = 2

Or partyid = 3

Or partyid = 4

Or partyid = 5

Or partyid = 6

Or partyid = 7

Or partyid = 8

Or partyid = 9

Or partyid = 10

partyid_strength How strongly ${q://QID147/ChoiceGroup/SelectedChoices} do you feel?

- Very strongly (1)
- Fairly strongly (2)
- Not very strongly (3)
- Don't know (4)

Page Break

Display This Question:

If partyid = 11

And And In politics, do you usually think of yourself as a(n): Text Response Is Not Empty

partyid_strength How strongly ${q://QID147/ChoiceTextEntryValue/2} do you feel?

- ○ Very strongly (1)
- ○ Fairly strongly (2)
- ○ Not very strongly (3)
- ○ Don't know (4)

End of Block: Party ID

Start of Block: Scales

risk On a scale of 0-10, where 0 means you are ‘completely unwilling to take risks’ and 10 means you are ‘very willing to take risks’ in general, how willing or unwilling are you to take risks?

	Completely unwilling to take risks	Very willing to take risks
	0 1 2 3 4 5 6 7 8 9 10	
1 ()		

Page Break

lr_scale In political matters, people talk of the 'left' and the 'right'. How would you place your views on this scale, generally speaking?

	Left						Right				
	0	1	2	3	4	5	6	7	8	9	10
1 ()											

Page Break

pol_system_t Timing
First Click (1)
Last Click (2)
Page Submit (3)
Click Count (4)

---

pol_system On a scale of 0-10, where 0 means you have ‘no confidence at all’ and a 10 means you have a ‘great deal of confidence’, how much confidence do you have in the following institutions?

	No confidence at all	Great deal of confidence
	0 1 2 3 4 5	6 7 8 9 10
The government ()		
Political parties ()		
Parliament ()		
The armed forces ()		
The press ()		
Television ()		
Labour unions ()		
The police ()		
The courts ()		
The civil service ()		
Universities ()		

Page Break

populism How much do you agree or disagree with the following statements?

	Strongly agree (1)	Somewhat agree (2)	Neither agree nor disagree (3)	Somewhat disagree (4)	Strongly disagree (5)
The politicians in the House of Commons need to follow the will of the people. (1)	○	○	○	○	○
The people, and not politicians, should make our most important policy decisions. (2)	○	○	○	○	○
The political differences between the elite and the people are larger than the differences among the people. (3)	○	○	○	○	○
I would rather be represented by a citizen than by a specialized politician. (4)	○	○	○	○	○
Elected officials talk too much and take too little action. (5)	○	○	○	○	○
What people call "compromise" in politics is really just	○	○	○	○	○

selling out on one’s principles. (6)

End of Block: Scales

Start of Block: covid19

c19_preamble In this part of the survey, you will be asked about your experience with the coronavirus (COVID-19) pandemic.

Page Break

had_c19 Have you been infected with coronavirus (COVID-19) since the start of the pandemic?

○ Yes (1)

○ No (2)

○ Unsure (3)

---

fam_c19 Besides you, has anyone in your household been infected with coronavirus (COVID-19) since the start of the pandemic?

○ Yes (1)

○ No (2)

○ Unsure (3)

Page Break

vaccinated_c19 Have you received a coronavirus (COVID-19) vaccine? Please do not include any information about booster shots received. We will ask about booster shots later.

- Yes, I have received a one-shot vaccine. (1)
- Yes, I have received the first dose of a two-shot vaccine. (2)
- Yes, I have received two doses of a two-shot vaccine. (3)
- No, I have not received any vaccine doses. (4)

Page Break

Display This Question:

If vaccinated_c19 = 4

no_vaccine_c19 You selected ‘No, I have not received any vaccine doses’ in the previous step. Please select all reasons that apply to your answer.

- ☐ A coronavirus (COVID-19) vaccine was not available to me. (1)
- ☐ I was not eligible for a coronavirus (COVID-19) vaccine. (2)
- ☐ I did not want to receive a coronavirus (COVID-19) vaccine. (3)
- ☐ I did not have time to access a coronavirus (COVID-19) vaccine. (4)

Page Break

Display This Question:

If vaccinated_c19 != 4

booster_c19 Have you received a coronavirus (COVID-19) booster vaccine?

○ Yes, I have received one or more shots of a coronavirus (COVID-19) booster vaccine. (1)

○ No, I have not received a coronavirus (COVID-19) booster vaccine. (2)

Page Break

Display This Question:

If booster_c19 = 1

booster_second How many booster shots have you received?

- ◯ I have not received a booster shot. (1)
- ◯ 1 booster shot. (2)
- ◯ 2 booster shots. (3)
- ◯ 3 booster shots. (4)
- ◯ 4 booster shots. (5)
- ◯ More than 4 booster shots. (6)

Page Break

Display This Question:

If booster_c19 = 2

no_booster_c19 You selected ‘No, I have not received a coronavirus (COVID-19) booster vaccine’ in the previous step. Please select all reasons that apply to your answer.

- ▢ A coronavirus (COVID-19) booster was not available to me. (1)
- ▢ I was not eligible for a coronavirus (COVID-19) booster. (2)
- ▢ I did not want to take a coronavirus (COVID-19) booster. (3)
- ▢ I did not have time to access a coronavirus (COVID-19) booster. (4)

Page Break

actions_om_c19_1 On a scale of 0-10, where 0 means 'never' and 10 means 'always', how frequently do you currently engage in the following actions or behaviours in response to the coronavirus (COVID-19)?

	Never Always Not Applicable<br>0 1 2 3 4 5 6 7 8 9 10
Wear a face mask in indoor public spaces. ()	
Wear a face mask in outdoor public spaces. ()	
Work, or attend school, from home. ()	
Avoid going to pubs/bars/restaurants. ()	
Social distance in indoor public areas. ()	

Page Break

actions_om_c19_2 On a scale of 0-10, where 0 means 'never' and 10 means 'always', how frequently do you currently engage in the following actions or behaviours in response to the coronavirus (COVID-19)?

	Never					Always			Not Applicable		
	0	1	2	3	4	5	6	7	8	9	10
Avoid small indoor gatherings (less than 6 people). ()											
Avoid large indoor gatherings (6 or more people). ()											
Social distance in outdoor public areas. ()											
Avoid small outdoor gatherings (less than 6 people). ()											
Avoid large outdoor gatherings (6 or more people). ()											

Page Break

vaccine_preamble Now you will be asked about your attitudes and beliefs towards vaccination and the coronavirus (COVID-19). There are no ‘right’ or ‘wrong’ answers. Please answer as honestly as you can.

Page Break

trust_vaccines On a scale of 0-10, where 0 means ‘strongly disagree’ and 10 means ‘strongly agree’, how much do you agree or disagree, with the following statements?

	Strongly disagree	Strongly agree
	0 1 2 3 4 5 6 7 8 9 10	
Vaccination is generally good for building immunity against the novel coronavirus that causes COVID-19. ()		
A vaccine will protect those who receive it from possible health effects of coronavirus (COVID-19). ()		
Without a vaccine, I am likely to catch coronavirus (COVID-19). ()		
I am worried about potential side effects of a coronavirus (COVID-19) vaccine. ()		
Receiving a coronavirus (COVID-19) vaccine has improved my sense of belonging to my community. ()		
Too much fuss is being made about the risk of coronavirus (COVID-19). ()		

Page Break

ignore_community On a scale of 0-10, where 0 means ‘strongly disagree’ and 10 means ‘strongly agree’, how much do you agree or disagree with the following statement?

	Strongly disagree	Strongly agree
	0 1 2 3 4 5 6 7 8 9 10	
Politicians usually ignore my community. ()		

Page Break

lives_over_liberty On a scale of 0-10, where 0 means ‘strongly disagree’ and 10 means ‘strongly agree’, how much do you agree or disagree with the following statement?

	Strongly disagree	Strongly agree
	0 1 2 3 4 5 6 7 8 9 10	
Saving lives is more important than personal liberty. ()		

Page Break

trust_vaccines2 Generally speaking, would you say that coronavirus (COVID-19) vaccines can be trusted?

- Yes, coronavirus (COVID-19) vaccines can be trusted. (1)
- No, coronavirus (COVID-19) vaccines cannot be trusted. (2)

Page Break

trust_who Which of the following sources would you trust MOST to help you decide whether you would get a COVID-19 vaccine and/or the booster?

- ◯ Your doctor or healthcare provider (1)
- ◯ Your co-worker (2)
- ◯ Your employer (3)
- ◯ Prime Minister Boris Johnson (4)
- ◯ Your local public health authority (5)
- ◯ Your friends or family (6)
- ◯ Your neighbours (7)
- ◯ Your local community (8)
- ◯ Your pastor, priest, or other religious leader (9)
- ◯ Other (please specify) (10) ____

End of Block: covid19

Start of Block: Media consumption

covid_news_source What is your primary source for news on the coronavirus (COVID-19) pandemic?

- ○ Newspapers (websites or in print) (1)
- ○ Television news (2)
- ○ Radio (3)
- ○ Social media (4)
- ○ WhatsApp (5)
- ○ WeChat (6)

Page Break

covid_news_volume How often have you read, listened to, or watched news related to the coronavirus (COVID-19) pandemic over the past week?

- Several times a day (1)
- Daily (2)
- Almost every day (3)
- A few times (4)
- Once (5)
- Never (6)

End of Block: Media consumption

Start of Block: Screen2

screen2 People are very busy these days and many do not have time to follow what goes on in the government. We are testing whether people read questions. To show that you've read this much, answer both "Extremely interested" and "Very interested":

- ☐ Extremely disinterested (1)
- ☐ Very disinterested (2)
- ☐ Somewhat disinterested (3)
- ☐ Neither disinterested nor interested (4)
- ☐ Somewhat interested (5)
- ☐ Very interested (6)
- ☐ Extremely interested (7)

End of Block: Screen2

Start of Block: conjoint

conjoint_preamble_t Timing
First Click (1)
Last Click (2)
Page Submit (3)
Click Count (4)

---

conjoint_preamble We are interested in knowing what you think the government should do about COVID-19 vaccines.<div>
</div><div>Recently boosters are being offered by public health authorities to citizens to increase protection against the novel coronavirus and COVID-19. </div><div>
</div><div>Imagine that, later in the year, your government will introduce a new vaccine policy that promotes boosters to protect the public from getting COVID-19 from new variants of the coronavirus. </div><div>
</div><div>To gauge your views, we will show you several pairs of hypothetical government policies about COVID-19 vaccines. For each pair of hypothetical vaccine policies, please think about which vaccine policy you prefer. There are no ‘right’ or ‘wrong’ answers. Please answer as honestly as you can. </div><div><div></div></div>

Page Break

conjoint1_t Timing
First Click (1)
Last Click (2)
Page Submit (3)
Click Count (4)

mobile1 If you are using your mobile phone for this survey, please turn the phone sideways to see the whole table.

conjoint1 <table class="UserTable"> <tbody> <tr> <td> </td> <td>Vaccine Policy A</td> <td>Vaccine Policy B</td> </tr> <tr> <td>How will booster appointments be scheduled?</td> <td> ${e://Field/schedule}</td> <td>${e://Field/schedule1}</td> </tr> <tr> <td>Will you get reminders to receive the booster?</td> <td>${e://Field/reminders}</td> <td>${e://Field/reminders1}</td> </tr> <tr> <td>Will employers be allowed to require that employees must receive the booster?</td> <td>${e://Field/mandate}</td> <td>${e://Field/mandate1}</td> </tr> <tr> <td>Will the government issue fines if you do not receive your booster when eligible?</td> <td>${e://Field/fines}</td> <td>${e://Field/fines1}</td> </tr> </tbody> </table>

prefer Which is your preferred choice of future vaccine policy?

○ Vaccine Policy A (1)

○ Vaccine Policy B (2)

support On a scale of 0-10, where 0 means you 'definitely do not support' this vaccine policy, and 10 means you 'definitely support' this vaccine policy, how would you rate each vaccine policy?

Definitely do not support						Definitely support				
0	1	2	3	4	5	6	7	8	9	10

Vaccine Policy A ()	
Vaccine Policy B ()	

Page Break

conjoint2_t Timing
- First Click (1)
- Last Click (2)
- Page Submit (3)
- Click Count (4)

---

mobile2 If you are using your mobile phone for this survey, please turn the phone sideways to see the whole table.

---

conjoint2 <table class="UserTable"> <tbody> <tr> <td> </td> <td>Vaccine Policy A</td> <td>Vaccine Policy B</td> </tr> <tr> <td> <p dir="ltr">How will booster appointments be scheduled? </p> </td> <td> ${e://Field/schedule2}</td> <td>${e://Field/schedule3}</td> </tr> <tr> <td>Will you get reminders to receive the booster?</td> <td>${e://Field/reminders2}</td> <td>${e://Field/reminders3}</td> </tr> <tr> <td>Will employers be allowed to require that employees must receive the booster?</td> <td>${e://Field/mandate2}</td> <td>${e://Field/mandate3}</td> </tr> <tr> <td>Will the government issue fines if you do not receive your booster when eligible? </td> <td>${e://Field/fines2}</td> <td>${e://Field/fines3}</td> </tr> </tbody> </table>

---

prefer2 Which is your preferred choice of future vaccine policy?

- ○ Vaccine Policy A (1)
- ○ Vaccine Policy B (2)

---

support2 On a scale of 0-10, where 0 means you 'definitely do not support' this vaccine policy, and 10 means you 'definitely support' this vaccine policy, how would you rate each vaccine policy?

	Definitely do not support						Definitely support				
	0	1	2	3	4	5	6	7	8	9	10

Vaccine Policy A ()	
Vaccine Policy B ()	

Page Break

conjoint3_t Timing
  First Click (1)
  Last Click (2)
  Page Submit (3)
  Click Count (4)

---

mobile3 If you are using your mobile phone for this survey, please turn the phone sideways to see the whole table.

---

conjoint3 <table class="UserTable"> <tbody> <tr> <td> </td> <td>Vaccine Policy A</td> <td>Vaccine Policy B</td> </tr> <tr> <td> <p dir="ltr">How will booster appointments be scheduled?</p> </td> <td> ${e://Field/schedule4}</td> <td>${e://Field/schedule5}</td> </tr> <tr> <td>Will you get reminders to receive the booster?</td> <td>${e://Field/reminders4}</td> <td>${e://Field/reminders5}</td> </tr> <tr> <td>Will employers be allowed to require that employees must receive the booster?</td> <td>${e://Field/mandate4}</td> <td>${e://Field/mandate5}</td> </tr> <tr> <td>Will the government issue fines if you do not receive your booster when eligible? </td> <td>${e://Field/fines4}</td> <td>${e://Field/fines5}</td> </tr> </tbody> </table>

---

prefer3 Which is your preferred choice of future vaccine policy?

○ Vaccine Policy A (1)

○ Vaccine Policy B (2)

---

support3 On a scale of 0-10, where 0 means you ‘definitely do not support’ this vaccine policy, and 10 means you ‘definitely support' this vaccine policy, how would you rate each vaccine policy?

Definitely do not support										Definitely support
0	1	2	3	4	5	6	7	8	9	10

Vaccine Policy A ()	
Vaccine Policy B ()	

Page Break

tradeoff_t Timing
First Click (1)
Last Click (2)
Page Submit (3)
Click Count (4)

tradeoff <div>Imagine the following scenario: In October of 2022, a new variant emerges which, like Omicron, is highly contagious. New vaccine boosters are developed. These boosters provide ${e://Field/effective} protection against infection from the new variant.</div><div>
</div><div>The government in your country would like individuals to take this booster shot. Which of the following policies would you support?</div>

	Yes (3)	No (4)	Unsure (5)
No one should be forced to take the booster, but it should be available to anyone who wants it. (1)	○	○	○
Those who do not take the booster should be stopped from entering any indoor public spaces (e.g., restaurants, entertainment venues) and/or using public transport. (5)	○	○	○
Employers should require their employees to get the booster. (6)	○	○	○
Those who do not take the booster should be fined by the government. (7)	○	○	○

End of Block: conjoint

Start of Block: screens3

screen3 Most modern theories of decision making recognize that decisions do not take place in a vacuum. Individual preferences and knowledge, along with situational variables can greatly impact the decision process. To demonstrate that you’ve read this much, just go ahead and select both red and green among the alternatives below, no matter what your favourite colour is. Yes, ignore the question below and select both of those options.<div>
</div><div>What is your favourite colour?
</div>

- ▢ White (1)
- ▢ Black (2)
- ▢ Red (3)
- ▢ Pink (4)
- ▢ Green (5)
- ▢ Blue (6)

End of Block: screens3

Start of Block: treatment1

timer1 Timing
- First Click (1)
- Last Click (2)
- Page Submit (3)
- Click Count (4)

---

preamble1 Imagine this: In October 2022, COVID-19 cases are rising in your area. The government is making another vaccine booster shot freely available to you as winter is approaching. <div>
</div><div>In this scenario, the government leaves it to every adult living in your country to choose whether they should get this vaccine booster shot or not. If you want a booster, you will have to call your local clinic to schedule a booster appointment.</div><div><div></div></div>

---

annual1 In this scenario, how likely is it that you would get this booster?

- Very likely (1)
- Somewhat likely (2)
- Slightly likely (3)
- Slightly unlikely (4)
- Somewhat unlikely (5)
- Very unlikely (6)

Display This Question:

If children != 4

And children , 4 Is Displayed

child1 In this scenario, how likely is it that you would allow your child/children under the age of 18 to get this booster?

- Very likely (1)
- Somewhat likely (2)
- Slightly likely (3)
- Slightly unlikely (4)
- Somewhat unlikely (5)
- Very unlikely (6)

Display This Question:

If parent_screen = 2

Or children = 4

child_all_1 In this scenario, if you had a child under the age of 18, how likely is it that you would allow your child to get this booster?

- Very likely (1)
- Somewhat likely (2)
- Slightly likely (3)
- Slightly unlikely (4)
- Somewhat unlikely (5)
- Very unlikely (6)

---

approve1 Do you approve or disapprove of the government’s action in this scenario? Please answer using the scale below, where 0 means ‘I disapprove of the government’s action’ and 10 means ‘I approve of the government’s action’.

	I disapprove of the government's action	I approve of the government's action
	0 1 2 3 4 5 6 7 8 9 10	
()		

---

action1 In this scenario, do you think the government is doing too little, just the right amount, or too much to manage the coronavirus (COVID-19) pandemic in your country?

- Too little (1)
- Just the right amount (2)
- Too much (3)

Page Break

manicheck_1_t Timing
First Click (1)
Last Click (2)
Page Submit (3)
Click Count (4)

manicheck_1 In this scenario, what did the government do to manage rising COVID-19 cases in your area?

- ◯ The government leaves it to every adult living in your country to choose whether they should get this vaccine booster shot or not. (1)
- ◯ The government announces that every adult living in your country will be automatically enrolled to receive this vaccine booster shot at a local clinic. (5)
- ◯ The government announces that every living adult in your country will be required to receive this vaccine booster to travel. (6)
- ◯ The government announces that it will fine adults living in your country who do not receive this vaccine booster. (7)

End of Block: treatment1

Start of Block: treatment2

timer2 Timing
First Click (1)
Last Click (2)
Page Submit (3)
Click Count (4)

preamble2 Imagine this: In October 2022, COVID-19 cases are rising in your area. The government is making another vaccine booster shot freely available to you as winter is approaching. <div>
</div><div>In this scenario, the government announces that every adult living in your country will be automatically enrolled to receive this vaccine booster shot at a local clinic. Your local clinic will call you to schedule a booster appointment at a convenient date and time. You can opt out of this automatic enrolment if you wish.<div> </div></div>

---

annual2 In this scenario, how likely is it that you would get this booster?

- ◯ Very likely (1)
- ◯ Somewhat likely (2)
- ◯ Slightly likely (3)
- ◯ Slightly unlikely (4)
- ◯ Somewhat unlikely (5)
- ◯ Very unlikely (6)

Display This Question:

If children != 4

And children , 4 Is Displayed

child2 In this scenario, how likely is it that you would allow your child/children under the age of 18 to get this booster?

- ○ Very likely (1)
- ○ Somewhat likely (2)
- ○ Slightly likely (3)
- ○ Slightly unlikely (4)
- ○ Somewhat unlikely (5)
- ○ Very unlikely (6)

Display This Question:

If parent_screen = 2

Or children = 4

child_all_2 In this scenario, if you had a child under the age of 18, how likely is it that you would allow your child to get this booster?

- ○ Very likely (1)
- ○ Somewhat likely (2)
- ○ Slightly likely (3)
- ○ Slightly unlikely (4)
- ○ Somewhat unlikely (5)
- ○ Very unlikely (6)

---

approve2 Do you approve or disapprove of the government’s action in this scenario? Please answer using the scale below, where 0 means ‘I disapprove of the government’s action’ and 10 means ‘I approve of the government’s action’.

	I disapprove of the government's action	I approve of the government’s action
	0 1 2 3 4 5 6 7 8 9 10	
()		

action2 In this scenario, do you think the government is doing too little, just the right amount, or too much to manage the coronavirus (COVID-19) pandemic in your country?

- ○ Too little (1)
- ○ Just the right amount (2)
- ○ Too much (3)

Page Break

manicheck_2_t Timing
First Click (1)
Last Click (2)
Page Submit (3)
Click Count (4)

---

manicheck_2 In this scenario, what did the government do to manage rising COVID-19 cases in your area?

○ The government leaves it to every adult living in your country to choose whether they should get this vaccine booster shot or not. (1)

○ The government announces that every adult living in your country will be automatically enrolled to receive this vaccine booster shot at a local clinic. (5)

○ The government announces that every living adult in your country will be required to receive this vaccine booster to travel. (6)

○ The government announces that it will fine adults living in your country who do not receive this vaccine booster. (7)

End of Block: treatment2

Start of Block: treatment3

timer3 Timing
First Click (1)
Last Click (2)
Page Submit (3)
Click Count (4)

---

preamble3 Imagine this: In October 2022, COVID-19 cases are rising in your area. The government is making another vaccine booster shot freely available to you as winter is approaching. <div>
</div><div>In this scenario, the government announces that every adult living in your country will be automatically enrolled to receive this vaccine booster shot at a local clinic. Your local clinic will call you to schedule a booster

appointment at a convenient date and time. You can opt out of this automatic enrolment if you wish.
</div>

---

*

text3 Please think about the government's actions in this scenario. Do you think this approach is appropriate? Do you think this approach will work for you? In at least one or two sentences, please write down your thoughts.

____

____

____

____

____

---

annual3 In this scenario, how likely is it that you would get this booster?

- ○ Very likely (1)
- ○ Somewhat likely (2)
- ○ Slightly likely (3)
- ○ Slightly unlikely (4)
- ○ Somewhat unlikely (5)
- ○ Very unlikely (6)

Display This Question:

If children != 4

And children , 4 Is Displayed

child3 In this scenario, how likely is it that you would allow your child/children under the age of 18 to get this booster?

- Very likely (1)
- Somewhat likely (2)
- Slightly likely (3)
- Slightly unlikely (4)
- Somewhat unlikely (5)
- Very unlikely (6)

Display This Question:

If parent_screen = 2

Or children = 4

child_all_3 In this scenario, if you had a child under the age of 18, how likely is it that you would allow your child to get this booster?

- Very likely (1)
- Somewhat likely (2)
- Slightly likely (3)
- Slightly unlikely (4)
- Somewhat unlikely (5)
- Very unlikely (6)

approve3 Do you approve or disapprove of the government’s action in this scenario? Please answer using the scale below, where 0 means ‘I disapprove of the government’s action’ and 10 means ‘I approve of the government’s action’.

	I disapprove of the government’s action	I approve of the government’s action
	0 1 2 3 4 5 6 7 8 9 10	
()		

action3 In this scenario, do you think the government is doing too little, just the right amount, or too much to manage the coronavirus (COVID-19) pandemic in your country?

- ○ Too little (1)
- ○ Just the right amount (2)
- ○ Too much (3)

Page Break

manicheck_3_t Timing
First Click (1)
Last Click (2)
Page Submit (3)
Click Count (4)

manicheck_3 In this scenario, what did the government do to manage rising COVID-19 cases in your area?

- The government leaves it to every adult living in your country to choose whether they should get this vaccine booster shot or not. (1)
- The government announces that every adult living in your country will be automatically enrolled to receive this vaccine booster shot at a local clinic. (5)
- The government announces that every living adult in your country will be required to receive this vaccine booster to travel. (6)
- The government announces that it will fine adults living in your country who do not receive this vaccine booster. (7)

End of Block: treatment3

Start of Block: treatment4

timer4 Timing
First Click (1)
Last Click (2)
Page Submit (3)
Click Count (4)

preamble4 Imagine this: In October 2022, COVID-19 cases are rising in your area. The government is making another vaccine booster shot freely available to you as winter is approaching. <div>
</div><div>In this scenario, the government leaves it to every adult living in your country to choose whether they should get this vaccine booster shot or not. If you want a booster, you will have to call your local clinic to schedule a booster appointment. </div>

---

*

text4 Please think about the government's actions in this scenario. Do you think this approach is appropriate? Do you think this approach will work for you? In at least one or two sentences, please write down your thoughts.

____

____

____

____

____

---

annual4 In this scenario, how likely is it that you would get this booster?

- ○ Very likely (1)
- ○ Somewhat likely (2)
- ○ Slightly likely (3)
- ○ Slightly unlikely (4)
- ○ Somewhat unlikely (5)
- ○ Very unlikely (6)

Display This Question:

If children != 4

And children , 4 Is Displayed

child4 In this scenario, how likely is it that you would allow your child/children under the age of 18 to get this booster?

- Very likely (1)
- Somewhat likely (2)
- Slightly likely (3)
- Slightly unlikely (4)
- Somewhat unlikely (5)
- Very unlikely (6)

Display This Question:

If parent_screen = 2

Or children = 4

child_all_4 In this scenario, if you had a child under the age of 18, how likely is it that you would allow your child to get this booster?

- Very likely (1)
- Somewhat likely (2)
- Slightly likely (3)
- Slightly unlikely (4)
- Somewhat unlikely (5)
- Very unlikely (6)

---

approve4 Do you approve or disapprove of the government’s action in this scenario? Please answer using the scale below, where 0 means ‘I disapprove of the government’s action’ and 10 means ‘I approve of the government’s action’.

	I disapprove of the government’s action	I approve of the government’s action
	0 1 2 3 4 5 6 7 8 9 10	
()		

action4 In this scenario, do you think the government is doing too little, just the right amount, or too much to manage the coronavirus (COVID-19) pandemic in your country?

- Too little (1)
- Just the right amount (2)
- Too much (3)

Page Break

manicheck_4_t Timing
First Click (1)
Last Click (2)
Page Submit (3)
Click Count (4)

manicheck_4 In this scenario, what did the government do to manage rising COVID-19 cases in your area?

- The government leaves it to every adult living in your country to choose whether they should get this vaccine booster shot or not. (1)
- The government announces that every adult living in your country will be automatically enrolled to receive this vaccine booster shot at a local clinic. (5)
- The government announces that every living adult in your country will be required to receive this vaccine booster to travel. (6)
- The government announces that it will fine adults living in your country who do not receive this vaccine booster. (7)

End of Block: treatment4

Start of Block: post-treatment

moregovernment_t Timing
First Click (1)
Last Click (2)
Page Submit (3)
Click Count (4)

moregovernment What else would you like the government to do to manage the coronavirus (COVID-19) pandemic in the scenario described above? <div>
</div><div>In at least one or two sentences, please write down your thoughts in the text box below.</div>

____

____

Page Break

post_t Timing

- First Click (1)
- Last Click (2)
- Page Submit (3)
- Click Count (4)

post There is a great deal of uncertainty about how the future might look with the coronavirus still in circulation. Please describe what you expect to happen with the coronavirus pandemic in 2022 in the text box below.

____

____

____

____

____

Page Break

future_scenario_1_t Timing

- First Click (1)
- Last Click (2)
- Page Submit (3)
- Click Count (4)

---

future_scenario_1 Next, we'd like to know what you think will happen in the future with the coronavirus (COVID-19). On a scale of 0-10, where 0 means ‘completely unlikely to happen’ and 10 means ‘completely likely to happen’, please tell us how likely you think the following will happen in the United Kingdom. <div>
</div><div>There are no ‘right’ or ‘wrong’ answers. Please answer as honestly as you can. </div>

	Completely unlikely to happen — Completely likely to happen<br>0 1 2 3 4 5 6 7 8 9 10
Compared to today, the number of COVID-19 related fatalities per day in the UK will be higher in 12 months. ()	
Compared to today, the number of COVID-19 cases per day in the UK will be higher in 12 months. ()	
COVID-19 vaccines available in the UK in 12 months will be more effective than the vaccines available today. ()	
People in the UK will be legally required to show proof of vaccination to access indoor public places in 12 months. ()	
Compared to today, public compliance with COVID-19 safety measures recommended by the UK government will be higher in 12 months. ()	
In 12 months, the UK government will recommend at least one new booster shot. ()	

Page Break

future_scenario_2_t Timing

- First Click (1)
- Last Click (2)
- Page Submit (3)
- Click Count (4)

---

future_scenario_2 Again, we'd like to know what you think will happen in the future with the coronavirus (COVID-19). On a scale of 0-10, where 0 means ‘completely unlikely to happen’ and 10 means ‘completely likely to happen’ please tell us how likely you think the following will happen in the United Kingdom. <div>
</div><div>There are no ‘right’ or ‘wrong’ answers. Please answer as honestly as you can.<div> <div> </div> </div></div>

	Completely unlikely to happen — Completely likely to happen<br>0 1 2 3 4 5 6 7 8 9 10
In 12 months, COVID-19 booster shots will be mandatory in the UK. ()	
There will be more restrictions on international travel to and from the UK in 12 months. ()	
Compared to today, people in the UK will socialise more in-person in 12 months. ()	
People in the UK will be legally required to wear masks in indoor public places in 12 months. ()	
Compared to today, people in the UK will work more often from home in 12 months. ()	

End of Block: post-treatment
